# Hidden Semi-Markov Models to Segment Reading Phases from Eye Movements

**DOI:** 10.16910/jemr.15.4.5

**Published:** 2022-09-30

**Authors:** Brice Olivier, Anne Guérin-Dugué, Jean-Baptiste Durand

**Affiliations:** Univ. Grenoble Alpes, Inria, CNRS, Grenoble INP, LJK, Inria Grenoble Rhone-Alpes, France; Univ. Grenoble Alpes, CNRS, Grenoble INP, GIPSA-Lab, 38000 Grenoble, France

**Keywords:** Eye movement, eye tracking, scanpath, reading, individual differences, hidden semi-Markov chains, segmentation

## Abstract

Our objective is to analyze scanpaths acquired through participants achieving a reading task aiming at answering a binary question: Is the text related or not to some given target topic? We propose a data-driven method based on hidden semi-Markov chains to segment scanpaths into phases deduced from the model states, which are shown to represent different cognitive strategies: normal reading, fast reading, information search, and slow confirmation. These phases were confirmed using different external covariates, among which semantic information extracted from texts. Analyses highlighted some strong preference of specific participants for specific strategies and more globally, large individual variability in eye-movement characteristics, as accounted for by random effects. As a perspective, the possibility of improving reading models by accounting for possible heterogeneity sources during reading is discussed.

## Introduction

The study of cognitive processes at stake in reading tasks is a major field of investigations in cognitive psychology and educational sciences (30; 31). To achieve this goal, eye tracking is a particularly useful and powerful source of information (8). Eye trackers provide almost straightforward access to the time sequence of read syllables and thus, words, sentences and full texts. On the one hand, this becomes fundamental material to explore and test hypotheses on mechanisms underlying processes at stake in semantic integration [occurring in reading tasks]. On the other hand, all data and knowledge accumulated has allowed the development of models describing the control of eye movements during reading. The most popular models are EZ Reader (34; 33), SWIFT (13; 27) and Glenmore (Reilly & Radach, 2002; 2006). These models provide theoretical frameworks to understand word identification, i.e. the lexical processing of words by the allocation of attention with the eye movements. Such models can predict when (fixation duration) and where (fixate or skip the next word) to move eyes. One major difference among them is the early stage of attention allocation assuming a serial lexical process for EZ-reader or a parallel one for consecutive words for SWIFT and Glenmore. But in all cases, these models assume a normal reading strategy called "rauding" (combination of reading and auding involving language comprehension) in the classification proposed by (5; 6), who introduced the terminology.

Until recently, experiments in eye tracking during reading tasks were restricted to carefully controlled experimental designs, particularly regarding textual materials. A possible reason for this is the heterogeneous nature of the reading process. It has been shown (Carver, 1997; Simola et al., 2008; 35) that depending on the reader’s current focus and intention, this process goes through different phases, such as text scanning or careful reading. In the case of multimedia documents, phases may also consist in making a connection between a text and an image or a video. The assumption of readers using personal and various strategies can be formulated from the observation of two scan paths from a same text read by two subjects, such as the ones in Figures S1 and S2 in Supplementary file. In Figure S1, fixations from 1 to 29 are on each word successively or nearly so, while each fixation after the 30th returns backward in the text, suggesting a change in the reading strategy. Every significant word but three of them were fixed. In Figure S2, fixations from 1 to 11 are on each word successively or nearly so, while fixations after the 12th alternate between long progressions (thus skipping some words) and backward fixations, suggesting once again a change in the reading strategy, but now using a different strategy for both readers after the first change. Six significant words were not fixed. Such heterogeneity precludes any straightforward statistical analysis based on global indicators computed at the whole scanpath scale (mean reading speed, saccade amplitude, fixation duration…), since this would mask the specific distributions associated to each phase. The ability to detect such phases to identify which one is currently carried out and what their dynamics are, is thus of significant importance to explain and analyse eye movements. The aim of our study is to propose a model to infer phases in scanpaths with similar statistical properties, where phase changes correspond to using a different reading strategy. Each phase is defined by a label, so that phases with a same label should be the expression of a same cognitive step reached during text processing. What is at stake is not only to infer underlying cognitive processes that explain the phases, but also to align or resynchronise parts of scanpaths from different readers or reading experiments, so as to obtain robust estimations of within-phase statistical properties.

Phase identification would offer new possibilities for analysing more complex reading scenarios, which are closer from real tasks of everyday’s life. Among those are for example, journal reading and web browsing for information search, in which readers have the possibility at every moment to decide to continue, to quit reading, or to change their focus of interest, etc. In this perspective, the ZuCo database consists of several datasets on natural reading of sentences from Wikipedia with different tasks such as reading or reading and evaluating semantic relation (21).

As a further consequence of reading phases, the topic of interest for researchers does not only focus on the reading processes, but also on the intertwined process of associated decisions on what to read next and how, closely linked to semantic integration and reader’s aims (15; 16). In this work, our hypothesis is that such phase changes exist in poorly constrained experimental reading situations, are latent and can be deciphered by appropriate statistical analysis of eye-movement data. Phases can be obtained using segmentation methods, such as hidden Markov models (HMMs). Segmentation consists in splitting scan paths into homogeneous portions (referred to as segments), in terms of eye-movement statistical properties.

HMMs are generally dedicated to modelling processes subject to regime switchings that separate successive phases, by associating one or several states to each possible phase. Not only do they provide signal segmentations but they also offer the possibility to model state dynamics, since in contrast to instantaneous (so called change-point) detection, probabilistic properties of segment durations and transitions to previously-visited states are included into the model. They have been used successfully to model the dynamics of eye movements, both in reading tasks - as previously mentioned - and in exploration of images. Functions of underlying Markovian cognitive states were introduced in (19) in a conceptual context of sequential problem solving. HMMs were then used in (Salvucci & Goldberg, 2000) to segment scanpaths into sequences of fixations and saccades. Two states represent fixations and saccades, identified from observed velocities in eye movements. Chuk and collaborators (2014) developed a Matlab® toolbox^1^ for eye-movement analysis with HMM. This time series model was first applied to face exploration in order to represent the evolution of eye positions into different regions of interest in the face (the hidden states). The classification of each individual HMM showed a dichotomy between holistic and analytic strategies of exploration. In (9), HMMs were used for task classification from scanpaths with a comparable setting as in (Chuk et al., 2014), except that the HMM has a third state associated to the centre bias. Considering more complex tasks involving intertwined cognitive processes, switching hidden Markov model was introduced to extract the cognitive states (the hidden states) from eye movements to analyse theirs transitions (7). Pairs of faces were shown to participants who had to indicate which face they preferred. An HMM-analysis of their eye movements aimed at capturing cognitive state transitions, highlighting an exploration period and a preference period, where the gaze is driven by the participant’s preference (which were the two model states) and providing predictions regarding times to decisions (7). In (25), HMMs were used to summarize the amount of eye exploration, once again from the sequence of eye positions on the image but using up to 14 states, which do not have any definite interpretation (the number of states was determined statistically using some information criterion).

HMMs were introduced in the context of reading tasks characterized by eye movements by (Simola et al., 2008). Three states (interpreted as scanning, reading and decision) were identified from sequences of saccade directions. In Simola’s study, HMMs mainly aimed at providing a probabilistic model for whole time series to perform their supervised classification, where the states accounted for regime switching over time.

In the context of analysing reading experiments, different classes of reading behaviours were defined and studied by (Carver, 1990). These classes were defined a priori in terms of tasks, mostly characterized by associated reading speeds of participants performing tasks. The comparison with emerging states inferred from eye tracking in free reading experiments is still an open question. Here, we address the problem with HMMs, which are relevant to identify phases in scanpaths in an unsupervised way, with homogeneous eye-movement dynamics within a phase and heterogeneous dynamics from a segment to another. HMMs simultaneously allow the clustering of similar segments into a labelled phase. Our approach, although based on the same statistical models, is different from the one by (Simola et al., 2008) since they used discriminative HMMs. As a consequence, their inferred reading states were defined so as to maximise discrepancies between models associated with three pre-defined tasks (word search, answering a question and search for the most interesting title within a collection).

In our study, we propose to use hidden semi-Markov chains (HSMCs) to infer states that optimize predictions of eye movements in less constrained experimental conditions. The Markovian assumption is relaxed in favour of a semi-Markov assumption to precisely model the number of steps (fixations in the case of this study) spent within each phase. Our estimation method simply maximises the fit between model and data. Our states are primarily defined by reading dynamics characterized by the number of words crossed in outgoing saccades, interpreted in terms of progression, regression, refixation, etc. This number of words is a signed value: positive in the case of progressions, negative in the case of regressions and null in case on refixations. This is also a difference with the approach proposed by Simola and collaborators (2008), who based their HMMs on several variables that depend on text layout, such as saccade directions and amplitudes. An unwanted consequence of this choice is that the states do not only reflect changes in the reading process but also changes in the text layout. In contrast, our approach is based on a single layout-independent variable. Moreover, after estimating the states from this variable only, we fully characterised them using saccade durations, directions and fixation durations, which integrate oculomotor features.

## Methods

Participants, textual material and the experimental procedure were the same as in (15). For the data sets, we used only in this study the eye tracking datasets but not the EEG datasets in the original files.

### Participants

Twenty-one healthy adults participated in the experiment, all French native speakers. Data of six participants were discarded because they did not follow the rules of the experiment thoroughly, misunderstood the task, or because data was too noisy or subjected to experimental errors during the acquisition with the eye tracker. The fifteen remaining participants (6 women and 9 men aged from 20 to 32 years, 25 years 9 months ± 7 years 6 months, mean plus or minus standard deviation, *sd*) had normal or corrected-to-normal vision. There were free of any medical treatment or any neurological or psychiatric disorder, past or present. None of them had prior experience with the experimental task. All gave their written and informed consent prior to the experiment and were paid 20€ for their participation. The whole experiment was reviewed and approved by the ethics committee of Grenoble CHU (“Centre Hospitalier Universitaire”) (RCB: n° 2011-A00845-36).

### Materials

180 short texts were extracted from the French newspaper Le Monde, edition 1999. Texts were given a topic and were constructed around three types, those which were highly related “HR” to the topic, or moderately related “MR” to the topic, or unrelated “UR” to the topic. There were 60 texts of each type, hence 180 in total. The semantic relatedness of the text to the topic was controlled by Latent Semantic Analysis (LSA) (10). To do so, LSA was trained on a French corpus of 24 million words composed of all articles published in the newspaper Le Monde in 1999 and a word or set of words (sentence, text, etc.) was represented by a vector in this 300-dimension semantic space. The number of dimensions *k* = 300 was determined in an empirical way by different tests (26). A very small number of dimensions results in an information loss and a very high number of dimensions does not allow one to make emerge the semantic relationships between the words (37). A cosine function was used to compute the similarity between vectors composed for the topic in the one hand and for the text in the other hand. The higher the cosine value, the more related the topic and the text are. For all highly related topics, semantic similarity with the text was above 0.2, while for all unrelated topics, semantic similarity was below 0.06. The moderately related texts were in-between. In the original study (15) from which the data for this article were derived, participants' text classification rates were as expected, namely a high acceptance rate for HR texts (92.9%), a chance-level acceptance rate for MR texts (47.2%), and a high rejection rate for UR texts (94.8%). The three text types HR, UR and MR reflect how texts were built but for a more detailed analysis of scan paths, some further distinction between HR text is introduced a posteriori in Subsection “Statistical Analysis”.

All the texts were composed of an average of 5.18 ± 0.7 (mean plus or minus standard deviation) sentences and 30.1 ± 2.9 words. The average number of characters of words was 5.34 ± 3.24. For the screen layout, the average number of lines was 5.18 ± 0.68, and the text was displayed with 40.1 ± 5.4 characters per line.

### Procedure

The goal of the experiment was to assess as soon as possible during reading whether the text was or not related to a given topic (so called target topic).

First the topic was presented to participants and then they clicked to start the trial. Then a fixation cross was presented on the left of the first character at the first line, to stabilize the eyes’ locations at the beginning of the text. The duration of this step was set at random between 700 and 900 ms to avoid anticipation of the reading start. Participants also did not know whether the text was HR/MR/UR so that they could not plan on a search strategy in advance. The texts were randomly ordered for each participant. When the text was displayed, participants read and had to mouse-click as fast as possible to stop reading and then had to decide during another screen if the text was related or not to the topic. Trials were repeated for the 180 texts with two breaks in-between.

### Apparatus

Each text was displayed at the centre of a 24-inch screen with a resolution of 1 024 by 768 pixels. Participants were seated 68 cm in front of the screen. Thus, texts covered in average 21° × 11° of visual angle and each character covered 0.52°of horizontal visual angle, corresponding to about 3.8 characters in fovea. Positions for both eyes on screen were recorded using a remote binocular infrared eye tracker EyeLink 1000 (SR Research) with a sampling rate of 1000Hz. Only positions of the guiding eye were analysed. Saccades and fixations were automatically detected by EyeLink software, based on three different thresholds: a minimum distance of 0.1° from the previous eye position, a minimum velocity of 30 °/s, and a minimum acceleration of 8 000 °/s^2^. A 9-point calibration was done every sixty trials. A drift correction was performed before each trial. Extra calibrations were performed if the participant was not able to stabilize the eye positions of the fixation cross or if the drift error was too large.

### From eye fixation to words and to reading strategies

During trials, the eye tracker gave the position of each fixation on the screen, and the fixation duration. The minimum (respectively maximum) fixation duration threshold was set to be 80ms (respectively 1 000 ms). All fixations outside these limits (4.8% in the population of 15 participants) were removed for all analyses. Fixations between lines or outside the text zone were also removed, leading to a removal rate of 0.3% from the initial set of fixations. Finally as each fixation was associated to its outgoing saccade, systematically the last fixation of each trial was not considered (5.4%).

A posteriori it was necessary to know which word was being processed by the participant. First, the word identification span was defined as the necessary area from which a word can be identified. This span varies according to the direction of the reading, the alphabet, or the language, but can also be micro-context related as it was for several reading models such as EZ-Reader (Reichle et al., 2003) or the SWIFT model (13). For simplicity, we used a fixed span that is considered for most of Latin languages (30): an asymmetrical window of 4 characters left and 8 characters right to the fixation, with a 35-pixel height. Moreover, a word may not entirely be located in the word identification span. Based on Farid and Grainger (14), we considered a word to be processed if at least 1/3 of its beginning or 2/3 of its end was inside the window. This result was obviously language sensitive, only valid in French, and considers that the important root of the word necessary to its understanding is located at the beginning of the word. Finally, another hypothesis had to be made on the processed word within the window since several words might be captured. For this, we assumed that only one word could be processed during a given fixation and that this word was chosen as the closest to fixation centre, excluding stop words. Consequently, one word per fixation was selected. Thanks to this data enrichment, features characterizing the reading strategy were defined.

From now throughout the article, the term “saccade” will be referred to the outgoing saccade of a given fixation. Thus finally, data associated with each fixation were the fixation duration, the fixed word, the saccade amplitude expressed in visual degree, the number of crossed words between two saccades and the saccade duration. We use “crossed” instead of “skipped” in this article since in some cases, words were not actually fixed by readers since they could infer these words without fixing them, while “skipped” would rather mean they intentionally ignored the semantic contents of a whole set of words. The saccade as a marker of the reading strategy was characterized by this number of crossed words, which would be negative for a backward progression, null for a refixation or positive for a forward progression.

At a whole text scale, the reading speed is known to be a global marker of the reading (Carver, 1990). At that scale, it was simply measured by how far (in words) a reader can go in a text in how much time. Since our aim was to segment text according the reading strategy, reading speed had to be computed at a finer scale. At the saccade scale, reading speed was computed as the number of crossed words during the saccade plus one (the fixed word during the current fixation) divided by the current fixation duration and the saccade duration. A shortcoming of computing instantaneous speeds at the saccade scale is it large variability, since means are more variable when computed on smaller samples. Thus, computing instantaneous speeds at the scale of one fixation / saccade and averaging them along the whole scanpath is expected to be less robust than dividing the total number of words fixed in a scanpath by its total duration. As a consequence from our hypothesis of various existing reading strategies, we had to compute reading speeds at an intermediate level. Within a given text segment, reading speed was evaluated as the number of crossed words plus the number of not yet fixed words divided by the sum of the fixation durations and saccade durations. For a text, composed of the different segments with different sizes (number of fixations) but with the same reading strategy, reading speed was computed by the ratio of the number of words (fixed and crossed during saccades) summed over all segments divided by the sum of fixation durations and saccade durations over all segments with the same reading strategy. If some word was crossed several times during the same scanpath, it was counted only once in the total number of words.

### Statistical analysis

#### General overview

As a preliminary analysis, the effects of text type on different reading characteristics were assessed using regression models. These models included Gaussian subject random effects to assess variability between subjects. Depending on the nature of the dependent variable (continuous, binary, categorical), we used either linear mixed models (LMMs), binomial generalized linear mixed models (BGLMMs) or multinomial generalized linear mixed models (MGLMMs), respectively. Normality of residuals in LMMs was assessed using Shapiro-Wilk normality tests complemented with histograms of empirical residuals. We investigated on the following effects: effects of text type (HR/MR/UR) on number of fixations per scanpath, on fixation durations, on saccade amplitude in degrees, on reading speed and on the number of crossed words (after categorization, leading to a so called *Read mode* variable defined hereafter). Significance of fixed effects within a given model was determined by ANOVAs. Model selection regarding fixed effects was achieved by computing BIC for each possible model built from a subset of covariates and their interactions. Model selection regarding fixed effects was achieved by computing BIC for each possible model built from a subset of covariates and their interactions. BIC (24) is composed by the difference between a model complexity term on the one hand, involving the number of model parameters, and on the other hand, some loglikelihood term quantifying the fit between the model and the data. The complexity term can be understood by considering that any model obtained as a generalization of another model necessarily fits any data set at least as well, even if it includes non-relevant effects. Thus, low BIC for a given model indicates a good fit of the data while keeping just relevant effects. We kept the set of covariates and interaction minimising BIC, meaning that the effects of covariates and interactions absent from that model could be ignored, from a statistical point of view. BIC for mixed models was defined as in (11). Confidence intervals on the standard deviation of random effects were obtained using profile likelihood as described in (2). In the case of MGLMMs, we used DIC instead of BIC (see 20).

To test the assumption of several reading strategies, we used an approach inspired by (Simola et al., 2008). The principle is to assume that at each time step *t* (each fixation), a reader follows some reading strategy represented by a state defined by the categorical variable *S_t_*. We do not observe the strategy explicitly; however switches in strategies can be deduced indirectly from observing the proportions of different types of eye movements that characterize strategies. To achieve this, we considered the number of words crossed in each outgoing saccade and categorised it into five different progression types, yielding a new variable denoted by *X_t_* and referred to as *Read mode*. Using the number of words crossed in each outgoing saccade makes *X_t_* invariant to changes in text layout, as opposed to saccade amplitudes and directions. Let us define and denote the five categories of *X_t_* as: “Fwd+” if the readers progress to more than one word forward, “Fwd” if they fix the word placed just after the previous word, “Rfx” if they fix the same word again, “Bwd” if they fix the word placed just before the previous word and “Bwd-” if they regress more than one word backward. Using the same strategies along successive fixations leads to statistically homogeneous zones regarding *X_t_*, referred to as segments (constant successive values of states *S_t_*, *S_t_*_+1_, …). The model depends on parameters estimated by maximum likelihood: the proportions of Fwd+, Fwd, Rfx, Bwd and Bwd- in each state, the probabilities to switch from current state to each possible state at next fixation (transition matrix) and the distributions of the number of fixations spent in each state (sojourn duration distribution). Interpreting the states as reading strategies relies on these parameters as well as external covariates (related to eye movements or to semantic contents). Segmentation of scanpaths, i.e., identifying successions of a same state, was performed to allow some statistical characterisation of states based on subjects or on external covariates.

The main steps of the HSMC analysis and their goals are summarized hereafter. The first two steps are related to modelling sequences of Read Modes, while the three last steps focus on the connexion between HSMC phases and other variables.

- We used information criteria: BIC (4) to select the number *K* of hidden states; we used state entropy (12) to compare different possible choices of *X_t_*.
- In some cases, we identified that states actually were a fine-scale decomposition of some more macroscopic state, defined as a pattern involving short cycles between the fine-scale states. In this case, states within these cycles were merged into a macroscopic state referred to as “phase” for the sake of interpretability. These phases were related to different reading strategies depending on their interpretation.
- To highlight between-participant variability in scanpaths, correspondence analysis (CA) (18) and an independence test were performed on the contingency table defined by the number of fixations in each phase for each subject. CA highlights associations between participants and phases in a graphical way.
- The effect of phase on reading speed was assessed using regression models, using the methodology presented at the beginning of Subsection “Statistical Analysis”. We also investigated the effect of text type on phase frequencies and the effect of text semantics on phase transitions (details provided hereafter).
- The software used for statistical analysis was VPlants, which is part of the OpenAlea platform (29), regarding HSMC analyses; the *lmer* package of the R software (Venables & Ripley, 2002) regarding LMMs, the *glmer* package in R regarding BGLMMs and the *MCMCGlmm* package in R (20) regarding MGLMMs.

#### Effects of semantics on transitions between phases

To ensure that phases have an interpretation as reading strategies and to investigate their relations with the semantic contents of texts, we assessed the effects of some words on phase transition probabilities. Our assumption was that participants took their decisions by detecting semantically related words to target topics (in HR texts) or incongruent words (in UR texts). This was highlighted by (15) on the same data set, showing that specific patterns arose in electroencephalograms, which could be interpreted as early markers for a positive decision in HR texts and a negative decision in UR texts. It was thus expected that such words triggered phase changes. This was addressed in our work by first detecting these words called “trigger words” and then, assessing the effect of distance to trigger words and of text types, on the probability of phase transitions. Due to the assumption of trigger words, the HR text type was refined, depending if at least one word of the target topic appeared or not in the text. In the positive case, the words that were both in the text and target topic were referred to as “target words” and such texts were referred to as “HR+”. In that case, trigger words necessarily include “target words”. In the negative case, the text did not contain “target words” and its type remained HR. The categorization including HR+ texts was referred to as “extended text type” hereafter.

Trigger words were detected using a FastText representation of words (23). This consists in embedding words into Euclidean spaces, allowing for computing semantic proximities between words using Euclidean metrics. Trigger words were the two closest words to target topic in HR / HR+ texts. In HR+ texts by definition, at least one word had cosine similarity 1. It was required in HR+ texts that the second closest word had minimal cosine similarity 0.3, otherwise only one “trigger word” was defined. It was required in HR texts that both closest words had minimal cosine similarity 0.3. Indeed, HR texts could have a very progressive semantic progression towards target topic, without clear trigger word. A threshold of 0.3 allowed to exclude these situations: HR scanpaths where all fixed words had cosine similarity less than 0.3 were ignored. In UR texts, trigger words corresponded to the two furthest words to target topic. Finally in MR texts, the two trigger words corresponded to the closest word and to the furthest word to target topic (no required bounds on cosine similarity).

Since HSMC states are random and hidden, the times of transitions are uncertain. Thus, instead of considering transition or not at trigger words, the effect of distance of transitions to trigger words was measured in number of fixations. For each state transition, the distance to closest “trigger word” was considered. Its effect of transition probabilities was assessed using regression models, with a linear assumption on the mapping between distance and frequencies, which was checked a posteriori.

Regarding statistical significance of the effect of distance, since texts were rather short by construction, yielding rather low total number of fixations per text, the effect of small distances increasing transition probabilities could be credited to distances being necessarily small, even if transitions were drawn at random and independently from the positions of “trigger” words. To assess this possible bias, randomized procedures based on permutation tests were used.

#### Detailed model description and code

A more formal description of the statistical models, estimation or model selection procedures and alternative definitions of *X_t_* are provided in the Supplementary file. These details and also a discussion regarding the hidden Markov vs. hidden semi-Markov assumption were developed in (28).

Data and source code used for statistical analyses are available on Inria Gitlab https://gitlab.inria.fr/statify_public/jemr-ema. The repository contains the analyses performed with Python and R packages in Jupyter notebook format and the Singularity / Docker images required to run them. The data set is also publicly available from Zenodo.org with doi http://doi.org/10.5281/zenodo.4655840. The experiment was not pre-registered.

## Results

### Summary statistics on observed data

After visual inspection of all scanpaths, some of them were discarded if the drifts on gaze positions were too large, making it impossible to assign a word at each fixation, typically when the eye positions were in between text lines. Moreover, scanpaths with less than four fixations were removed (assumed to be non-characteristic of the task). Globally, HSMC models were run on 2 390 scanpaths with a total of 39 564 fixations.

Table 1 summarizes the average individual statistics per participant, on the number of scanpaths, number of fixations per text, fixation duration, saccade amplitude expressed in visual degree [°] or in number of crossed words during each saccade [w] and reading speed expressed in words per minute [wpm].

### Effect of text type on scanpath characteristics

#### Statistics before segmentation by HSMC

For each text type, the scanpaths were characterized by the number of fixations, fixation duration, saccade amplitude expressed in degrees [°] and in number of words [w] and reading speed (see Table 2).

Text type had a strong effect on the number of fixations per text (ANOVA highlighting significance at level 10^-16^ with chi-square statistics 298.0 on 2 degrees of freedom), with a strong individual variability (BIC difference of -877 with null model ignoring individual effects). Distributions per text type are represented in Figure S3 in Supplementary file. UR and HR texts did not show significant differences while MR texts had quite higher numbers of fixations. This was shown by a LMM, taking text type as a covariate with HR as a reference value. The 0.995 confidence intervals were (3.8, 5.7) for MR parameter and (-1.7, 0.2) for UR parameter. The sample sizes (number of HR, MR and UR scan paths) were 803, 785 and 802, respectively.

Text type had a strong effect on the fixation duration (ANOVA highlighting significance at level 10^-7^ with chi-square statistics 31.0 on 2 degrees of freedom), with a strong individual variability (BIC difference of -5004 with null model ignoring individual effects). Distributions per text type are represented in Figure S4 in Supplementary file. HR and MR texts did not show significant differences while UR texts had quite shorter fixation durations. This was shown by a LMM, taking text type as a covariate with HR as a reference value. The 0.995 confidence intervals were (-3.0, 1.3) for MR parameter and (-6.5, -2.0) for UR parameter. The sample sizes (number of fixations in HR, MR and UR scanpaths) were 12 316, 15 745 and 11 503, respectively.

Text type had some moderate effect on saccade amplitude in degrees (ANOVA highlighting significance at level 0.05% with chi-square statistics 15.1 on 2 degrees of freedom), with a strong individual variability (BIC difference of -1 080 with null model ignoring individual effects). Distributions per text type are represented in Figure S5 in Supplementary file. As shown by a LMM, taking text type as a covariate with HR as a reference value, 0.995 confidence intervals were (-0.00, 0.25) for MR parameter and (-0.26, 0.1) for UR parameter. Using 0.93 confidence intervals, the effects became significant. This suggested potentially higher amplitudes for MR texts and lower amplitudes for UR texts. The sample sizes (number of saccades in HR, MR and UR scanpaths) were 12 316, 15 745 and 11 503, respectively.

Text type had also a strong effect on reading speed (ANOVA highlighting significance at level 10^-16^ with chi-square statistics 204.6 on 2 degrees of freedom), with a strong individual variability (BIC difference of -1098 with null model ignoring individual effects). MR and HR texts did not show significant differences while UR texts had larger reading speeds, as illustrated in Figure S6 in Supplementary file. The LMM 0.995 confidence parameter was (-38.6, 3.6) for MR parameter and (64.1, 106.1) for UR parameter.

Normality tests indicated lack of normality of empirical residuals in models for number of fixations, fixation duration, saccade amplitude and reading speed at level 10^‑16^ (Shapiro-Wilks statistics of 0.89, 0.94, 0.85 and 0.97), presumably due to skewness in their distributions. However, the distributions were visually close to normal (see Figures S7, S8 and S10 in Supplementary file), except in the case of saccade amplitude, which seemed bimodal and very strongly skewed (see Figures S9 in Supplementary file).

MGLMMs modelling the effect of text type on *Read mode* showed significance of random individual effects, with 99.5% credibility intervals of (0.3, 2.6) for the variance for individual effect. This was confirmed by the large, negative difference (-570) in DIC values between models with and without random individual effects. The difference in DIC between the null model and the model with a text type effect was -547, indicating absence of effect for text type. This result was somehow counter-intuitive given the significance of all individual parameters at level 0.001, particularly regarding overrepresentation of Fwd+ in UR texts. This did not seem either in accordance with empirical distributions depicted in Figure S11 in Supplementary file. The *Read mode* frequencies per text type are summarized in Table 2. Detailed descriptions of estimates are provided in Supplementary file (Table S1 regarding this model).

#### HSMC modelling

**Figure 1.**
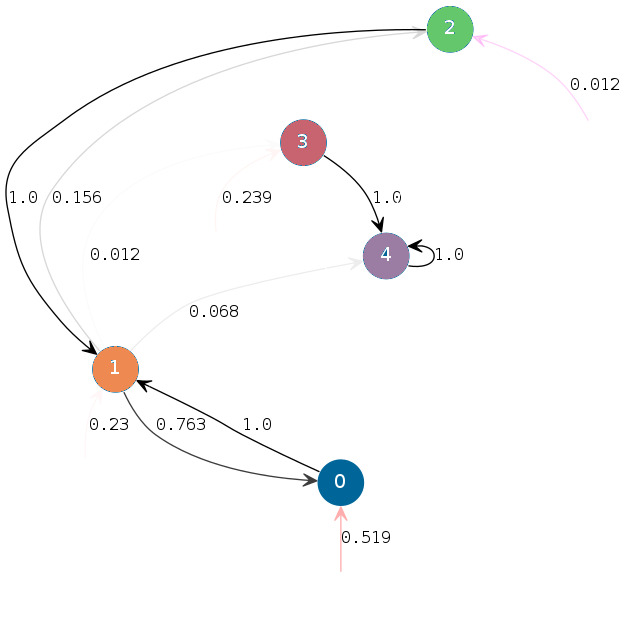
HSMC transition diagram. Vertices correspond to states and arcs, to transition probabilities above 0.01. Arcs in light grey have low transition probabilities. The initial state distribution is represented by pink arrows pointing to possible initial states but issued from no other state.

**Figure 2.**
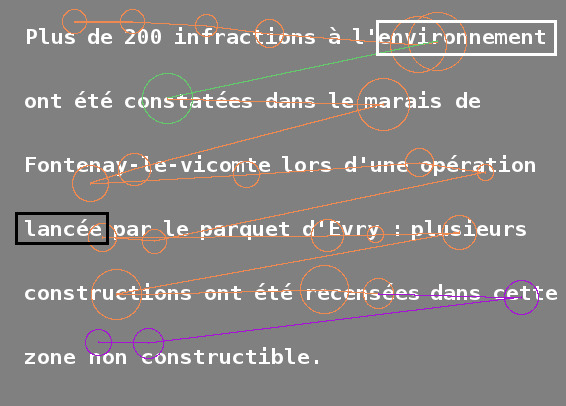
Scanpath of some MR text with phase restoration. Target topic is “Nuclear waste”. Phase 1 (normal reading) is in orange, phase 2 (information search) in green and phase 4 (slow confirmation) in purple. Translation: “More than 200 violations to environment were recorded in the swamp next to Fontenay-le-vicomte during an operation launched by Evry’s prosecution service; several buildings were recorded within this non-buildable land.” The word framed in white (“environment”) is the closest to target topic, that framed in black (“launched”) is the farthermost to target topic.

**Figure 3.**
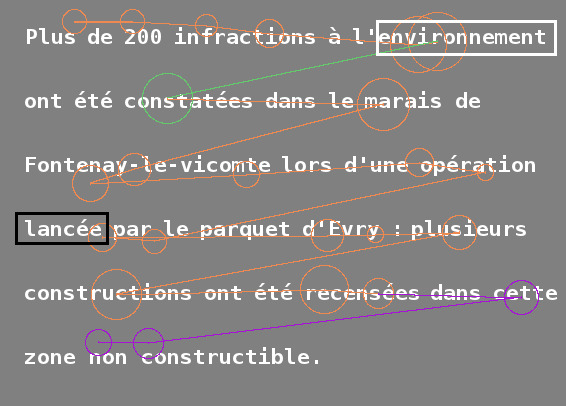
Scanpath of some HR+ text with phase restoration. Target topic is “Oil Price”. Phase 3 (fast reading) is in red and phase 4 (slow confirmation) in purple. Translation: “Algerian economy depends on the evolution of crude oil exchange rates. After they fell down to historically low levels, the announcement by producing countries of production reduction led to price recovery”. The words framed in white are the closest to target topic (“oil” and “crude”).

BIC selected a 5-state model. The estimated parameters and distributions are represented in Table 3 (see also Table S4 and Figure S12 in Supplementary file). State 0 was characterized by very short sojourn lengths and systematic alternation with state 1, which was typical of a macro-state, called here “phase”. Thus, phases were defined as Phase 1 = {State 0, State 1} and Phase *i* = {State *i*} if *i* >1. Initial phase probabilities were 0.75 for Phase 1 (sum of initial probabilities for states 0 and 1), 0.01 for Phase 2, 0.24 for Phase 3 and 0 for Phase 4. Phase 1 had intermediate probabilities for Fwd+, Fwd and Rfx (see Table 3). Thus it could be interpreted as the normal reading phase, abbreviated as “NR”. Phase 2 usually separated two runs of Phase 1 (see Figure 1) and its sojourn duration can be short: 3.48 fixations in average (see Table 3). Its interpretation was not obvious. It was characterized by high probabilities of Rfx, Bwd- and Fwd+ reflecting numerous discontinuities during a normal reading. Therefore we named it an information search phase “IS” characterized by many saccade orientation changes. Phase 3 was transitory, meaning that once left it could not be reached again. It had the highest Fwd+ probability and thus was interpreted as a fast reading phase “FR”. Phase 4 was absorbing, meaning that no other phase could be accessed from it (thus its sojourn time was infinite). It had the highest Bwd and Bwd- probabilities and was followed by no other phase. Thus it was interpreted as a slow confirmation phase “SC”. State restoration provides a visual interpretation of phases, as illustrated in Figures 2 and 3. Other scanpaths are provided in Figures S13 and S14 in Supplementary file to illustrate other typical behaviours.

The comparison between Figures 2 and 3 highlights that in FR, refixations appeared to be less frequent than in NR while in SC, backward fixations were more frequent than in both NR and SC. Note that every scanpath did not necessarily end in Phase 4 (SC) since decisions could be reached in any other phase and even before the end of the text was reached, as illustrated in Figure S11.

**Figure 4.**
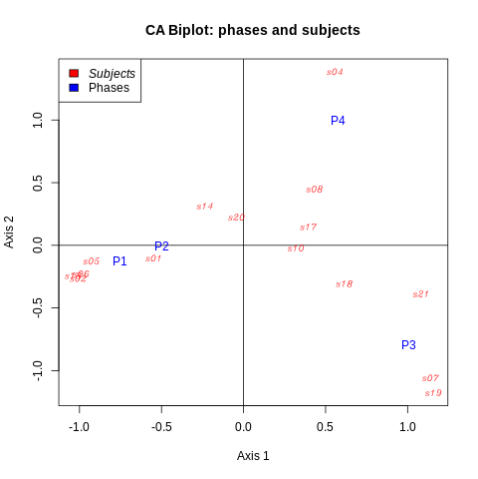
First principal plane of correspondence analysis. Phases 1 to 4 are represented by blue points labelled as P1 to P4. Participants 1 to 21 are represented by red points labelled as s01 to s21.

*Inter-individual variability of reading strategies.* There was some variability in the use of reading strategies (phases) among participants. This was highlighted by particular associations between participants and phase probabilities. An independence test between phase and participant yielded a test statistic of 2.1×10^5^ for 42 degrees of freedom, corresponding to very clear rejection of independence (the p-value cannot be computed since it was too close to 0). The first CA plane (linear space spanned by the first two principal components) is represented in Figure 4.

The ratio of preserved inertia was 99% in this plane. Three clusters of individuals were highlighted:

1) individuals using phases 1 and 2 (normal reading and information search) at the detriment of the other phases, e.g., Participant S02 at the left-hand part of Figure 4;
2) individuals using phase 3 (fast reading) primarily at the detriment of phase 1 and secondarily 4, e.g., Participants S19 at the bottom-right corner of Figure 4 (fast readers);
3) individuals using phase 4 (slow confirmation) primarily at the detriment of phase 1 and secondarily 3, e.g., Participant S04 at the upper-right corner of Figure 4 (careful readers).

Effect of phase on reading speed. To validate state interpretations systematically in terms of reading speed, the latter was computed for each phase. Mean reading speed was 304 words per minute (wpm) in NR, 183 wpm in IS, 509 in FR and 263 in SC, which is consistent with our former interpretation and with Figure S15 in Supplementary file. Linear mixed models were used to test the effect of phase and individual variability, accounting for already confirmed text type effects (see Subsection “Statistics before segmentation by HSMC”). The phase effect was assessed as significant by ANOVA at level 10^-16^ (with chi-square statistics 270.2 on 3 degrees of freedom and 3 555 segments), while with a BIC difference of -875 with the null model, individual variability was assessed as highly significant. The estimated standard deviations were 96 (individual) and 235 (residual), the 95% confidence interval for the individual standard deviation being (66, 140). The normality test indicated lack of normality of empirical residuals at level 10^-16^ (see Figure S16 in Supplementary file) with a Shapiro-Wilks statistics of 0.80).

**Figure 5.**
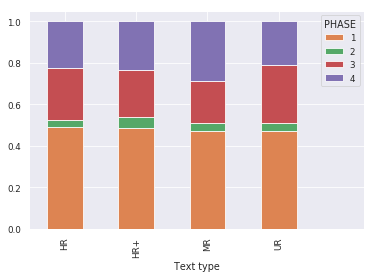
Phase sample distribution per extended text type. Phase 1 is normal reading, phase 2 is information search, phase 3 is fast reading and phase 4 is slow confirmation.

Effect of text types on phase. The state and phase sample distributions are represented in Figures S17a and S17b, respectively (see Supplementary file). The state and phase sample distributions per extended text type are represented in Figures S18 (Supplementary file) and 5, respectively. The effect of extended text type on phase distribution was assessed using MGLMMs (estimates are provided in Tables S2 and S3, see Supplementary file) The credibility interval at level 99.5% for the variance of individual effect was (0.8, 6.5), indicating high individual variability. This was consistent with the high difference in DIC values (‑13 173) between models with and without random individual effects. The difference in DIC values with the null model without text type effect was also large (-466), indicating a strong effect of text type. MR texts were characterized by less frequent use of phase 4 (SC) and less frequent use of phase 3 (FR), UR texts by less frequent use of phase 1 (NR) and more frequent use of phase 3 (FR) and HR texts by more frequent use of phase 1 (NR), which is visually consistent with Figure 5 despite the large individual variability.

**Figure 6.**
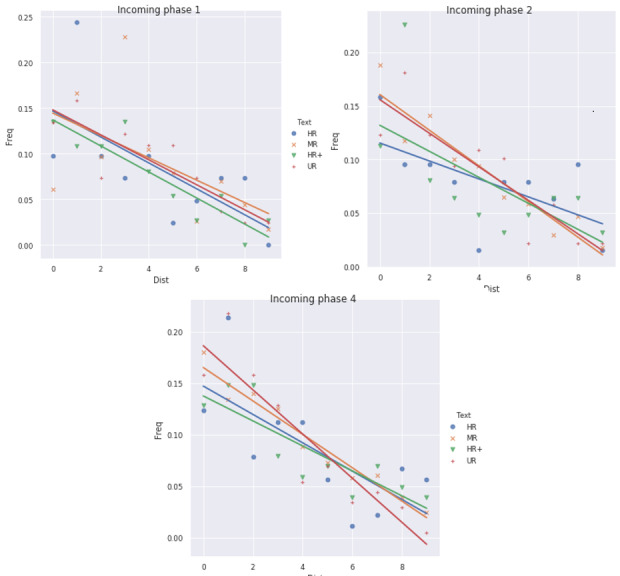
Relationship between distance to trigger words and frequencies of transitions arriving into phase 1 (normal reading, top left), 2 (information search, top right) and 4 (slow confirmation, bottom).

*Effect of phase trigger words on phase transitions.* The results related to the effect of trigger words are presented in Figure 6. Each diagram represents the distance (in number of fixations) between a transition and the closest trigger word (x-axis) together with the associated transition frequency (y-axis). The three diagrams correspond to different incoming phases (phase type following a transition). The regression line is shown for each extended text type. Transitions to phase 3 (FR) were too rare (See Figure S19 in Supplementary file) and thus their frequencies could not be reliably estimated. Examples of trigger words in specific scanpaths are illustrated in Figures 2 (MR text), 3 (HR+) and S13 in Supplementary file (UR).

It can be seen from Figure 6 that linear models are relevant to explain dependencies between distances and frequencies. Thus the effects of distance, extended text type and phase on frequencies were considered through LMMs. The model with order-3 interactions between distance, phase and text type minimized BIC (-787). The second lowest value of BIC was -780 (model with marginal effect of distance and interactions between phase and text type). The best model was compared with a linear model with the same structure of effects but no individual random effect, yielding a BIC difference of -42 (significant individual variability). The 95% confidence interval on standard deviation parameter was (0.05, 0.1), which confirmed significance of individual variability. The effect of each factor was tested individually, highlighting some very strong effects of phase and extended text type, as well as some significant effect of distance (BIC difference of -32, p-value in ANOVA 3×10^‑5^ using a chi-square test on 12 degrees of freedom, with sample size 849). BGLMMs were applied to binary variables corresponding to occurrence or not of transitions at a given distance to trigger words. However, estimation did not converge for several combinations of interactions, so we could not assess the effects of all interactions between covariates. Nonetheless, BGLMMs led to conclude to very strong marginal significance of the three effects.

Regression coefficients are interpreted as follows: strongly negative slopes correspond to transitions occurring more frequently around keywords. Lines had more negative slopes in UR texts, suggesting that incongruent words appeared to induce immediate changes in reading strategies. The slopes in MR texts had intermediate values between those of UR and HR/HR+ texts. The slopes in HR and HR+ were also negative and could not be assessed as different.

Randomized tests showed that random allocations of transitions yielded some lower difference in BIC with the null model than the true difference (-39) in 52% of simulated sequences in the case of constrained phase permutations and in 78% of simulated sequences in the case of free phase permutations (See BIC difference histograms in Figures S20 and S21 from Supplementary file). This shows that even if the effect of distance is removed, it remains assessed as statistically significant in a large proportion of simulations. This suggests that the effect of distance could be partly due to scanpath shortness. However, the same procedure applied to BGLMMs on absence / presence of transition led to in 0% BIC difference that were smaller in simulated sequences than the BIC difference in true data (-123), both in constrained and free phase permutations settings (See BIC difference histograms in Figures S22 and S23 from Supplementary file). Thus, the lack of significance of the effect of distance through permutation tests using LMMs could be due to some lack of model adequacy, as compared to BGLMMs, instead of being due to some real absence of effect.

## Discussion

Our methodology confirmed the importance of modelling phase changes for accurate interpretation of eye movements in loosely-controlled information search tasks. Phase interpretation was supported by contrasted characteristics in terms of sequencing during the task, *Read mode* frequencies, reading speeds and text semantics, summarized here with text types and trigger words.

Particularly, reading strategies were interpreted in terms of reading speeds using the *Read mode* variable, which was directly connected to HSMC parameters. It is however interesting to compare reading speeds obtained in each phase to those associated with Carver’s reading “gears” (1990): learning, rauding, scanning and skimming. The mean speed of 304 wpm in NR corresponded to rauding, the speed of 509 wpm in FR was intermediate between skimming and scanning, 263 wpm in SC was intermediate between rauding and learning, while the speeds of 183 wpm in IS was comparable with learning.

Although our study had somewhat different focuses and aims compared to the study by (Simola et al., 2008), the latter addressed related questions with related tools. In particular, the states they obtained could be compared with ours. Their study highlighted three states, which were stable in the three different tasks they considered. Three distinctive HMMs (one for each task, “word search”, “question-answer”, “true interest”) were embedded into a unique discriminative model in order to classify each observed trial, from eye-movement features, into one of the three classes, i.e. one of the three tasks. The model selection based on classification highlighted three hidden states for each model. Those states, called scanning, reading and decision were interpreted on the basis of the distribution of the input observed data, specifically on the fixation durations, saccade amplitudes and saccade directions. In our model, five states gathered into four phases were observed. When comparing the interpretations made by (Simola et al., 2008) and ours, there was a very simple matching between them: scanning could be assimilated with FR, decision with SC and reading with NR, while IS appeared to be more specific to our experiments.

Another significant output of this study was the predominant individual variability, which could be observed at every level of the analysis. Here again, HSMC models led to a precise characterization of this variability in terms of favoured use of contrasted reading strategies depending on each individual, leading to some clustering of the population (reader types): standard, fast and careful readers. Such a characterization of reader types is original, regarding other studies that summarize scan paths to global statistics, mainly: reading speed, saccade amplitude and fixation duration (17; Jarodzka et al., 2017; 36). Such studies may aim at using eye-tracking to assess the level of expertise of learners in different reading tasks. In this context, classical statistics do not account for intra- or sometime event inter- scan path variability and a same reading speed could be obtained by using different reading strategies (for example, fast reading combined with slow reading, or just normal reading). Using the notions of phase and transitions could lead to a better characterization and classification of reader types.

In our study, the individual variability was so high that probably, it partially or totally masked the effects of other factors. This is the case regarding the effect of text type on *Read mode*. This effect could not be confirmed statistically, which was somehow counter-intuitive given the significance of all individual parameters at level 0.001, particularly regarding overrepresentation of Fwd+ in UR texts. This did not seem either in accordance with empirical distributions depicted in Figure S11 in Supplementary file. Similarly, the effect of distances to trigger words on phase transitions was assessed as moderate by LMMs, while Figure 6 suggested some strong effect. This could be due to individual variability partly masking this effect.

This suggests, on the one hand, that accounting for individual variability in modelling is of uttermost importance and on the other hand, that some additional participants may have to be involved in the experiment so as to confirm the effects of text type and distance to trigger words on transition probabilities.

Comparisons between the three text types based on different indicators (reading speed, phase distribution and transitions, effect of trigger words, scanpath lengths) highlighted that UR (unrelated) texts were easy to process (more fast reading FR, less normal reading NR) whereas MR (moderately related) texts were more difficult, as expected (more slow confirmation SC, less FR). The difficulty of HR (highly related) texts was intermediate and no significant difference was found between HR and HR+ texts. In fact, for UR texts, it was less the semantic construction of the text as such that was relevant than the elaboration of the semantic similarity of the text with the displayed topic. This semantic similarity estimated in the LSA space was always very low whatever the scanpaths, because these texts contained words with low frequencies that were unrelated to their target topic. However, for MR texts the semantic construction had to contribute to their comprehension so as to be able to answer regarding the link with the topic. Finally, for HR texts, there was a strong variability in the construction of the semantic link between the topic and the read words, because this link depended on the presence or absence in the text of a word belonging to the topic. As a result, our study showed the possibility to obtain such characterization of the different text types by using just eye movements and a very rough description of the text semantic contents (summarized by distances to trigger words). It also showed that *Read mode*, despite its very straightforward definition, was sufficiently synthetic to reflect some major effects of interest in reading experiments.

The quantitative results of our study could be used to improve existing reading models such as EZ-Reader or SWIFT. Indeed, these models try to identify, through eye movements, the different phases in the reading process such as overall attention shifting and lexical decoding. Considering the EZ-reader model, there are two main assumptions. The first hypothesis states that attention is allocated serially on one word at a time and that attention is intrinsically linked to lexical processing. The second hypothesis states that eye-movement control and saccade control are decoupled. The model assumes that the lengths and the frequencies of words have a great importance for the lexical steps, from the earlier step, called “familiarity check” to the last step, called “completion of lexical access”. It is well known that the fixation duration on a word is a function of a range of linguistic factors and among these, word length and frequency are lexical variables with a large effect on fixation duration (30). For each word of the text, these two variables and the word predictability in the context of the text sentences are the core variables of the model (Rayner et al., 2004). From these input data, the model will provide for each word, the probability to be fixed and the fixation duration. But to estimate all the parameters of the model from known scanpaths during reading, it is necessary to assume that they come from the same reading strategy in the sense of the Carver's classification. Let us illustrate this idea for two configuration parameters of the EZ reader model, the minimum duration of the “familarity check” and the “systematic error”. The first one is the fixed part of the estimation of the duration of the “familarity check”. The variable part is indexed on the frequency and the predictibility of the words. It is expected that this minimal duration should depend on the level of comprehension depth induced by readers’ intentions, and also their linguistic expertise or their reading skills (3). This is also the case for the systematic error parameters determining the probability for eye movements to undershoot or overshoot their intended targets. Therefore, the scanpath segmentations obtained by our approach could be used to calibrate specific parameters in EZ-reader because these segmentations provided homogeneous statistical properties. Both models could then by coupled, so that the HMSC model could trigger parameter switches in EZ-reader when changing reading strategy.

Our approach considered mixed models to characterize the effect of eye-movement- or semantic-related covariates on phases. This in an improvement compared to (Simola et al., 2008), whose study, contrarily to ours, did not account for individual variability nor for the effects of semantic covariates. However from a methodological point of view, a possible weakness of our work remained in the separate use of HMSC models on the one hand, and GLMM modelling of the effect of phases including individual effects on the other hand. Indeed, individual variability was highlighted in state dynamics and emission distributions, so ideally, this would have to be accounted for in parameter estimation by maximum likelihood. Inference of state-based parameters based on MAP restoration was likely to cause biases since uncertainty on the state values was not accounted for in post-hoc analyses. In the same spirit, including effects of covariates (e.g., distance to trigger words or type of text in transition probabilities) could be integrated in HMSC models directly, by using GLMMs instead of plain distributions in the transition matrix, state sojourn duration and emission distributions. Inference in such models was studied in particular cases by (1). Another possibility to account for individual heterogeneity would be to resort to mixtures of HSMMs, but this would lead to some significant increase in the number of model parameters, whereas mixed effect models had tied parameters.

Although we developed some methodology to connect reading phases or strategies to text semantics, the latter was here summarized to two trigger words. The effect of their distance to phase transitions was assessed with LMMs, showing that lines had more negative slopes in UR texts. This suggests that incongruent words appeared to induce immediate changes in reading strategies. They would probably have a strong effect on the decision to stop reading and proceed to the answer, although this has not been assessed here. The slopes in MR texts had intermediate values between those of UR and HR/HR+ texts, suggesting that even the concept of trigger words in MR texts is ill-defined (the notion of MR text being even vague itself): Participants may base their decisions on either incongruent words or words that are related to target topic, to decide how to explore texts. The slopes in HR and HR+ were also negative and could be assessed as different, showing that reading words from the target topic has no stronger effect on strategy changes than reading words only close to the target topic.

Moreover, our analyses did not account for inhomogeneity of the semantic progression within different texts. In some of them, relevant information with respect to target topic was brought linearly while in some others, it was brought abruptly in one or two major steps. Some text clustering could reveal helpful to investigate connections between the dynamics of accumulated information, as quantified by FastText and the use of particular strategies.

Lastly, our approach opens new avenues to jointly analysing eye movements and electroencephalograms (EEGs). This would allow some characterisation of the brain connections that are activated or not in each reading strategy, thus confirming that the phases inferred from the HSMC model have an interpretation in terms of cognitive steps to solve the reading task. From a general point of view, performing analyses based on EEGs only is particularly difficult in free reading tasks. This is partly due to the high level of noise, related to both inter- and intra-individual variability. Another source of difficulty is the lack of synchronization of different individuals reading the same text using different strategies. Here, eye-movement based segmentation acts as a medium to resynchronize portions of scan paths coming from different individuals and trials. The reason for this is that segments of the same nature, with definite dates of beginning and ending, associated with synchronized EEG signals, may be assumed to have common features due to inherent homogeneity in a given phase. Performing within-segment analyses is thus expected to reduce heterogeneity and to facilitate identification of specific EEG patterns characterizing cognitive steps leading to decisions.

### Ethics and Conflict of Interest

The author(s) declare(s) that the contents of the article are in agreement with the ethics described in http://biblio.unibe.ch/portale/elibrary/BOP/jemr/ethics.html and that there is no conflict of interest regarding the publication of this paper.

### Acknowledgements

This work has been partially supported by the LabEx PERSYVAL-Lab (ANR-11-LABX-0025-01) funded by the French program “Investissement d’avenir”.

The authors acknowledge useful contributions by B. Lemaire regarding eye-movement and natural language processing. They acknowledge helpful remarks by S. Achard to improve the discussion and relevant suggestions from anonymous reviewers to improve the readability of the article, as well as the scope of results.

**Table 1.** Average ± between-participant standard deviation [within-participant standard deviation] for the number of texts, the number of fixations per text, the fixation duration, the saccade amplitude in [°] and in number of words [w], and the reading speed [wpm]

Feature	*Average ± between-sd [within-sd]*
Scan paths number	159.3 ± 22.4
Fixations number per text	16.6 ± 4.7 [7.1]
Fixation duration [msec]	184.0 ± 23.1 [62.3]
Saccade amplitude [°]	5.3 ± 0.67 [3.9]
Saccade amplitude [w]	1.9 ± 0.5 [2.5]
Reading speed [wpm]	404.9 ± 119.8 [155.8]

**Table 2.** Average ± between-participant standard deviation [within-participant standard deviation] for the number of fixations per text, the fixation duration, the saccade amplitude expressed in degree [°] and in number of words [w], and the reading speed [wpm] depending of the type of text, followed by *Read mode* frequencies and total number of fixations per text type.

	Text Type		
Feature	HR	MR	UR
Fixations number per text	15.4 ± 4.4 [6.6]	20.1 ± 5.0 [7.0]	14.3 ± 5.1 [6.0]
Fixation duration [msec]	185.8 ± 22.9 [63.0]	184.2 ± 22.8 [62.6]	181.9 ± 23.9 [61.0]
Saccade amplitude [°]	5.3 ± 0.7 [3.9]	5.4 ± 0.7 [3.9]	5.2 ± 0.6 [3.8]
Saccade amplitude [w]	1.8 ± 0.5 [2.2]	1.9 ± 0.5 [2.6]	2.0 ± 0.5 [2.5]
Reading speed [wpm]	381.9 ± 117.7 [124.8]	365.2 ± 99.1 [116.5]	466.8 ± 151.6 [183.9]
*Read mode*			
Long regression (Bwd-)	0.06	0.07	0.06
Regression (Bwd)	0.02	0.02	0.02
Refixation (Rfx)	0.26	0.26	0.25
Progression (Fwd)	0.23	0.21	0.22
Long progression (Fwd+)	0.42	0.43	0.43
Total number of fixations	12 316	15 745	11 503

**Table 3.** HSMC sojourn duration and emission probabilities per state. B is the shifted Binomial distribution and NB is the shifted Negative Binomial distribution. ∞ indicates an absorbing state. Emission probabilities are the probabilities of each possible Read mode value in each state and phase (NR for normal reading, IS for information search, FR for fast reading and SC for slow confirmation)

Phase	State	Sojourn duration		Emission probabilities distribution			
		Sojourn duration distribution	Mean	Long regression (Bwd-)	Regression (Bwd)	Refixation (Rfx)	Progression (Fwd)	Long progression (Fwd+)
NR	0	B	1.29	0.008	0.029	0.642	0.321	0.000
	1	B	1.22	0.004	0.012	0.026	0.242	0.715
IS	2	NB	3.38	0.017	0.000	0.384	0.000	0.444
FR	3	NB	13.37	0.030	0.025	0.109	0.254	0.583
SC	4	∞	∞	0.198	0.047	0.159	0.139	0.457

## supplementary material



## Footnotes

http://visal.cs.cityu.edu.hk/research/emhmm/
