# Supplementary material for: Hidden Semi-Markov Models to Segment Reading Phases from Eye Movements
Source: J Eye Mov Res. 2022 Sep 30;15(4):10.16910/jemr.15.4.5. doi: 10.16910/jemr.15.4.5 (PMC10292930; doi:10.16910/jemr.15.4.5)
Supplement: Supplementary file 1 [file jemr-15-04-e-SD1-01.pdf]

Hidden Semi-Markov Models to Segment Reading Phases from Eye Movements Supplementary files

Brice Olivier
Univ. Grenoble Alpes, Inria, CNRS, Grenoble INP, LJK,
Inria Grenoble Rhone-Alpes, France

Anne Guérin-Dugué
Univ. Grenoble Alpes, CNRS,
Grenoble INP, GIPSA-Lab, 38000
Grenoble, France

Jean-Baptiste Durand
Univ. Grenoble Alpes, Inria, CNRS,
Grenoble INP, LJK,
Inria Grenoble Rhone-Alpes, France

Detailed description of statistical analysis

At each fixation t within a scanpath, let V_t be the number of words crossed in one saccade. Let W_t be the signed number of words crossed in one saccade: in case of refixations W_t=V_t=0, in case of forward progressions W_t =V_t >0 and in case of backward progressions, W_t=-V_t <0. We now define X_t as follows (X_t is referred to as *Read mode*): X_t = W_t if W_t = -1, 0 or 1; X_t ="<-1" if W_t < -1; X_t =">1" if W_t > 1. In the sequel, W_t > 1, W_t = 1, W_t = 0, W_t = -1 and W_t < -1 will be referred to using the following abbreviations respectively: Fwd+, Fwd, Rfx, Bwd and Bwd-. Note that several other choices were possible to define X_t, which are detailed in Olivier et al. (2021). In this work we kept the choice for X_t, that minimized joint state entropy, as computed according to Durand and Guédon (2016).

The principle of hidden Semi-Markov chains (HSMCs) to perform scanpath segmentation is to associate observations X_t at each time step t (*Read mode* at fixation t) with some underlying (or "hidden") categorical random variable (state) S_t representing the current probability distribution of the states. Thus, successive identical states represent homogeneous segments in terms of these eye-movement properties. State transitions correspond to marked changes in the *Read mode* distribution. The distribution of *Read mode* within each state, potentially combined with external variables, may lead to state interpretation as reading strategies. HSMCs assume that the hidden state process - (successive values of states S_1, S_2, …) enters an initial state k with probability π_k, so that π_k is the probability that the state is k for the first fixation of the scanpath, characterised by *Read mode* X_1.

Once a state entered, the process stays in that state during a random number of fixations with distribution p_{θ_k}. When leaving state k, some new state l is entered with probability $A_{k,\ell}$. At time t if S_t=k, then X_t=x occurs with probability P(X_t=x | S_t=k)= $B_{k,x}$. Denoting by K the number of possible values for S_t=k, the set of parameters λ is thus defined by $\pi=(\pi_k)_k$, the transition matrix A, the observation probability matrix B and the parameters $(\theta_k)_k$ for the state duration distributions.

The HSMC parameters were estimated by maximum likelihood using the EM algorithm (see Yu, 2010). This is an iterative algorithm that aims at maximising the likelihood of data by building a sequence of parameters (estimates) with increasing likelihood values. It requires some initial guess of parameter λ. The estimate obtained after convergence may strongly depend on this initial guess, which is why we considered 100 initial random parameter values and kept the one that led to the estimate at convergence with largest likelihood.

The considered parametric distributions for the quantitative variables were uniform, Poisson, binomial and negative binomial with shift parameters in the four cases. For example if D is a random variable distributed as the usual binomial distribution B(n, p) with probability p, the shifted binomial B(s, n, p) is the distribution of $D+s$ where s is some (deterministic, unknown) additional shift parameter.

Since states are unknown (even after estimating λ), modellers cannot choose their values or interpretations. In practice one crucial tool to interpret parameters is matrix B. State k is mostly defined by the composition of state k in the different possible *Read mode* values, i.e., P(X_t=Bwd- | S_t=k), ..., P(X_t=Fwd+ | S_t=k). For example, if state k has a large probability P(X_t=Fwd+ | S_t=k) compared to P(X_t=Fwd+ | S_t=j) for the other states j, state k can be interpreted as a fast reading state, although even if that state P(X_t=Bwd- | S_t=k) > 0 so that regressions could occur occasionally with, say, a lower probability than if the state was j. The other main tool to interpret states is state restoration, which aims at finding a sequence of states values $S_1,...,S_T$ for a given scanpath summarized by the observed *Read mode* sequence of $X_1,...,X_T$, such that $S_1,...,S_T$ is the most probable state sequence given $X_1,...,X_T$ and λ. This so-called restored sequence, computed by the Viterbi algorithm (see Yu, 2010), actually provides the segmentation in homogeneous zones with respect to the distribution of X_t, such as the segmentations depicted in Figures S11 and S12 within this document. As a complement to interpret states, we can use the distributions of other covariates Y_t that have not been used to estimate λ (e.g., saccade amplitude, fixation duration and reading speed).

The number K of hidden states is unknown and has to be determined. Here we used BIC (Boucheron & Gassiat, 2007) to select K. BIC is defined by minus the loglikelihood value (after maximisation with respect to parameters) plus a positive term, referred to as penalty, that depends on the number of model parameter and the logarithm of the sample size. Since the maximum loglikelihood necessarily growths as K increases, the penalty is introduced to avoid overfitting. The penalty ensures that if the sample size is large enough and that one of the compared models is the right one, BIC will find it with a probability that tends to one (this principle extends to generalised linear mixed models hereafter). In practice, we set a maximal value K_{max} for K and for every value between K=1 and K= K_{max}, we estimated a model with K states and computed its BIC. We then kept the value of K with lowest BIC value, indicating an optimal trade-off between data fit and model complexity. Models were discarded if they yielded mean segment lengths shorter than 4 fixations or longer than 25 fixations, since these were either specific of one single subject or could not be interpreted as reading strategies. In some cases, we identified that states actually were a fine-scale decomposition of some macroscopic state defined as a pattern involving short cycles between the fine-scale states. For example, a fast alternation between states 0 and 1, which always occurred together was observed. In this case, these cycles were merged into macroscopic states referred to as "phases" for the sake of interpretability. This was only observed for states 0 and 1 which was merged to define phase 1. For the other states, the state and the associated phase were identical.

To highlight subject variability in scanpaths, correspondence analysis (CA) (Greenacre, 1984) and a chi-squared independence test were performed on the contingency table defined by the number of fixations in each phase for each subject, merging fixations from all scanpaths.

The effect of text type was assessed by tests in regression models. Three families of models were considered: linear mixed models (LMMs), binomial generalized linear mixed models (BGLMMs) and multinomial generalized linear mixed models (MGLMMs) with Gaussian random individual effects. LMMs were used to assess effects of covariates on quantitative variables (reading speed and fixation durations computed for each scanpath) using the *lmer* package of the R software (Venables & Ripley, 2002). Significance of fixed effects within a given model was determined by ANOVAs. BGLMMs were used to assess effects of covariates on binary variables (occurrence of phase transitions or not) using the *glmer* package in R. Model selection regarding fixed effects was achieved by computing BIC on the whole collection of models built from all subsets of covariates and their interactions. In practice, for each possible subset of covariates and interactions, we estimated that model and scored it with BIC. We kept the set of covariates and interaction minimising BIC, meaning that the effects of covariates and interactions absent from that model can be ignored, from a statistical point of view. The BIC for mixed models requires a specific definition to account for random effects and their variance possibly being equal to zero (at the boundary of the set of parameters). We used the adaptation proposed in (Delattre et al., 2014).

Significance of individual effects was assessed by comparing BIC values of the models with the best set of covariates, considering in turn variants with and without individual random effects. This was complemented by the use of confidence intervals on the standard deviation of random effects, using profile likelihood as described in (Bates et al., 2014). MGLMMs were used to assess the effect on nominal categorical variables (*Read mode* and Phase) using Bayesian estimation with the *MCMCGlmm* package in R (Hadfield, 2010), since no referenced package could estimate MGLMMs by maximum likelihood while providing confidence intervals and BIC values. Significance of individual effects was assessed using credibility intervals at level 0.995 on variance parameters. In the case of MGLMMs, we used DIC (Spiegelhalter et al., 2002) instead of BIC to assess significance of fixed effects. DIC is an information criterion such that penalises data fit by model complexity. BIC has a closed-form penalty, while the penalty in DIC is implicit. The model minimizing the considered criterion (either BIC or DIC, depending on models) was selected.

Consistently with the workflow describe above, the effect of text type (categorical variables HR, MR and UR) on the number of fixations per scanpath, fixation duration, saccade amplitude, reading speed, and *Read mode* frequencies were assessed using MGLMMs and the MCMCGlmm package in R. The effect on quantitative variables was assessed using LMMs. In both cases, random subject effects were included and their significance was tested. Effects of categorical predictors were tested using analyses of variance (ANOVAs). Normality of residuals in LMMs was assessed using Shapiro-Wilk normality tests complemented with histograms of empirical residuals.

"Trigger words" were detected using a FastText representation of words (Joulin et al., 2017). This consisted in embedding words into Euclidean spaces, allowing for computing semantic proximities between words using Euclidean metrics (here, the cosine distance). Only two "trigger words" per text were defined. Trigger words were the two closest words to target topic in HR / HR+ texts. In HR+ texts by definition, at least one word had cosine similarity 1. It was required in HR+ texts that the second closest word had minimal cosine similarity 0.3, otherwise only one "trigger word" was defined. It was required in HR texts that both closest words had minimal cosine similarity 0.3. Indeed, HR texts could have a very progressive semantic progression towards target topic, without clear trigger word. A threshold of 0.3 allowed to exclude these situations: HR scanpaths where all fixed words had cosine similarity less than 0.3 were ignored. In UR texts, trigger words corresponded to the two furthest words to target topic. Finally in MR texts, the two trigger words corresponded to the closest word and to the furthest word to target topic (no required bounds on cosine similarity).

Since HSMC states are random and hidden, the times of transitions are uncertain. Thus, instead of considering transition or not at trigger words, the effect of distance of transitions to trigger words was measured in number of fixations, focusing on trigger words with lowest distance to transitions. Its effect of transition probabilities was assessed using regression models. Firstly, frequencies for the distances associated to each incoming phase (among every possible distance for that phase) were modelled with linear mixed regressions, using distance, text type and phase as predictors, with subjects as random effects. Secondly, the binary random variable corresponding to occurrence or not of a transition at each possible distance of a fixation to trigger word was modelled with generalized linear mixed regressions. Binomial distributions were considered, using the canonical link function and the same three predictors as above. In both approaches, models with interactions of order 2 and 3 between predictors were estimated, in addition to models without interaction. Models were compared using BIC. The model with minimal BIC (referred to as M1) was then used to assess the effect of random subject effects, by comparing BIC with that of a model without random effects. M1 was also compared with the model obtained by removing distance as a predictor (referred to as M0). The justifications for using both approaches (linear models on frequencies or GLMMs on binary variables) were twofold: firstly, GLMMs easily suffer from lack of convergence for high-order interactions and thus some of these models cannot be compared. Secondly, the linear assumptions on frequencies seemed reasonable given the shape of the cloud of points (See Figure 6).

Since texts were rather short and total numbers of fixations were rather low (see Subsections "Materials" and "Summary statistics on observed data"), the effect of small distances increasing transition probabilities could be credited to distances being necessarily small, even if transitions were drawn at random and independently from the positions of trigger words. To assess this possible bias, randomized procedures were developed. In their spirits, these consisted in

reassigning random positions to trigger words, though in practice refined procedures were developed to preserve some phase structure in scanpaths. The first one consisted in sampling transition locations (uniformly without replacement) and permuting the order of phases, thus constraining the number of transitions to remain the same within each scanpath. The second one consisted in sampling the number of transitions with replacement within their empirical distribution and drawing phase values uniformly, with the constraint that successive phases must be different. In both cases, the whole data set was resampled 1 000 times. Each time M1 and M0 were estimated again, as well as their difference in BIC. The percentage of differences obtained by resampling was compared to the true difference, thus assessing the significance of the distance effect.

Detailed presentation of regression models

We present here the regression models selected at each step of the statistical analysis (meaning, after selection by some information criterion).

(Absence of) effect of text type on Read mode

Model:

$$\log \frac{P(X_{t,s,p} = x)}{P(X_{t,s,p} = 0)} = \beta_x + \zeta_s$$

where $X_{t,s,p}$ represents Read mode at position *p* within scanpath *s* with text type *t*.

Table S1. Posterior distribution of GLMM parameters β_x : posterior mean, left limit (l-95% CI) and right limit (r-95% CI) of a 95% credible interval, one minus level required for the credible interval to contain 0 (pMCMC)

Read mode	Posterior mean	l-95% CI	u-95% CI	pMCMC
Regression (Bwd)	-0.8952	-1.3109	-0.4422	<0.001
Refixation (Rfx)	2.4007	2.3111	2.4706	<0.001
Progression (Fwd)	2.0514	1.9688	2.1407	<0.001
Long progression (Fwd+)	2.8943	2.7729	2.9875	<0.001

The values for $var(\zeta_s)$ are: post.mean 0.7786, l-95% 0.314, CI u-95% 1.44. The article provides 99.5% credible intervals.

Effect of extended text type on phases

Model:

$$\log \frac{P(Y_{t,s,p} = r)}{P(Y_{t,s,p} = 0)} = \beta_r + \delta_{t,r} + \zeta_s$$

where $Y_{t,s,p}$ represents phase at position *p* within scanpath *s* with text type *t*.

Table S2. Posterior distribution of GLMM parameters β_r : posterior mean, left limit (l-95% CI) and right limit (r-95% CI) of a 95% credible interval, one minus level required for the credible interval to contain 0 (pMCMC). IS stands for Information Search, FR for Fast Reading and SC for Slow Confirmation.

Phase	Posterior mean	l-95% CI	u-95% CI	pMCMC
1 IS	-2.94173	-3.69958	-2.20122	<0.001
2 FR	2.23517	2.10716	2.37067	<0.001
3 SC	2.03218	1.90425	2.14628	<0.001

Table S3. Posterior distribution of GLMM parameters $\delta_{t,r}$: posterior mean, left limit (l-95% CI) and right limit (r-95% CI) of a 95% credible interval, one minus level required for the credible interval to contain 0 (pMCMC). IS stands for Information Search, FR for Fast Reading and SC for Slow Confirmation.

Extended text type	Phase	Posterior mean	l-95% CI	u-95% CI	pMCMC
HR+	1 IS	0.54492	0.37527	0.71244	<0.001
HR+	2 FR	-0.03767	-0.12424	0.04745	0.306
HR+	3 SC	0.11030	0.02819	0.19486	0.008
MR	1 IS	0.27842	0.16185	0.39390	<0.001
MR	2 FR	-0.10370	-0.16231	-0.05296	<0.001
MR	3 SC	0.41723	0.33974	0.49163	<0.001
UR	1 IS	0.41069	0.29705	0.54896	<0.001
UR	2 FR	0.27009	0.19537	0.32881	<0.001
UR	3 SC	0.09926	0.02076	0.18834	0.022

The values for $var(\zeta_s)$ are: post.mean 2.44, l-95% 0.95, CI u-95% 4.46. The article provides 99.5% credible intervals.

State	Sojourn duration			Emission distribution				
	Sojourn duration distribution	Mean	Standard dev.	Long regression (Bwd-)	Regression (Bwd)	Refixation (Rfx)	Progression (Fwd)	Long progression (Fwd+)
0	B(1,4,0.098)	1.29	0.515	0.008	0. 029	0. 642	0. 321	0. 000
1	B(1,5,0.056)	1.22	0.458	0. 004	0. 012	0.026	0. 242	0. 715
2	NB(1, 0.680, 0.215)	3.48	3.406	0. 017	0. 000	0. 384	0	0. 444
3	NB(1, 41.175, 0.769)	13.365	4.010	0.0302	0.0247	0.1084	0.2542	0.583
4	∞	∞	0	0.198	0.047	0.159	0.139	0.457

Supplementary table

Table S4. HSMC sojourn duration and emission probabilities per state. B(i, n, p) is the shifted Binomial distribution with shift parameter i, number of trials n and probability p. NB(i, q, p) is the shifted Negative Binomial distribution with shift parameter i, shape parameter n and probability p. ∞ indicates an absorbing state. Emission probabilities are the probabilities of each possible ReadMode value in each state.

Supplementary figures

Boxplots indicate the three quartiles of distributions and 1.5 interval quartile ranges of the lower and upper quartiles.

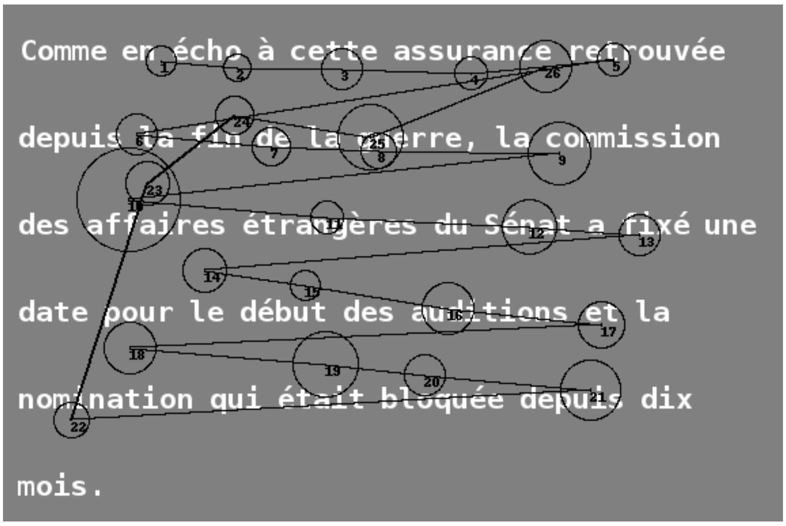

Figure S1. Scanpath of some text read by subject 8. Fixations are numbered by order. Translation: "As if echoing the self-confidence regained since the end of the war, the Foreign Affairs committee of Senate set up a date to begin auditions and to proceed to nomination, which had been blocked for ten months".

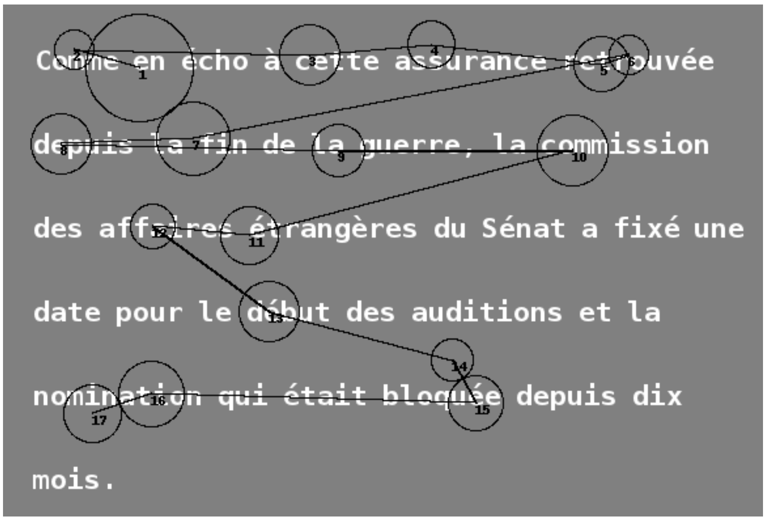

Figure S2. Scanpath of some text read by subject 14. Fixations are numbered by order.

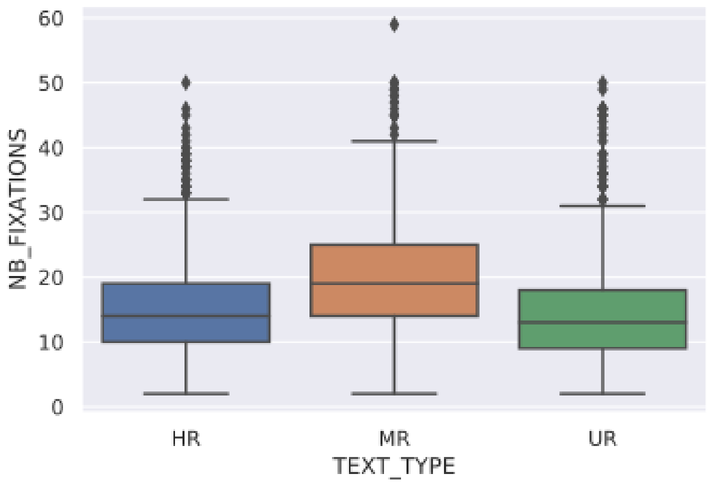

Figure S3. Boxplot of numbers of fixations (NB_FIXATIONS) for each text type.

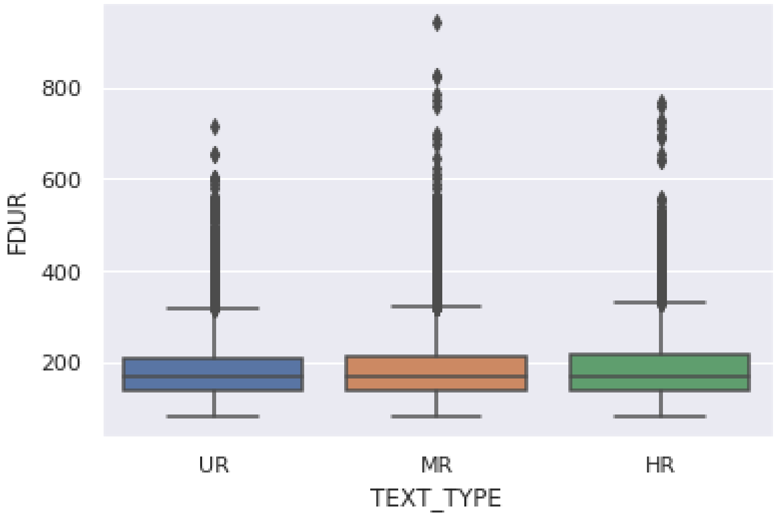

Figure S4. Boxplot of fixation duration (FDUR) in ms for each text type.

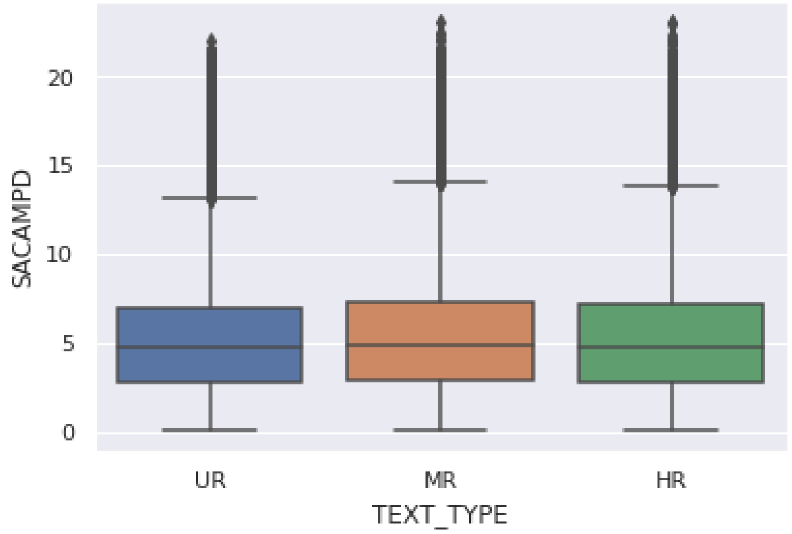

Figure S5. Boxplot of saccade amplitude (SACAMP) in degrees for each text type.

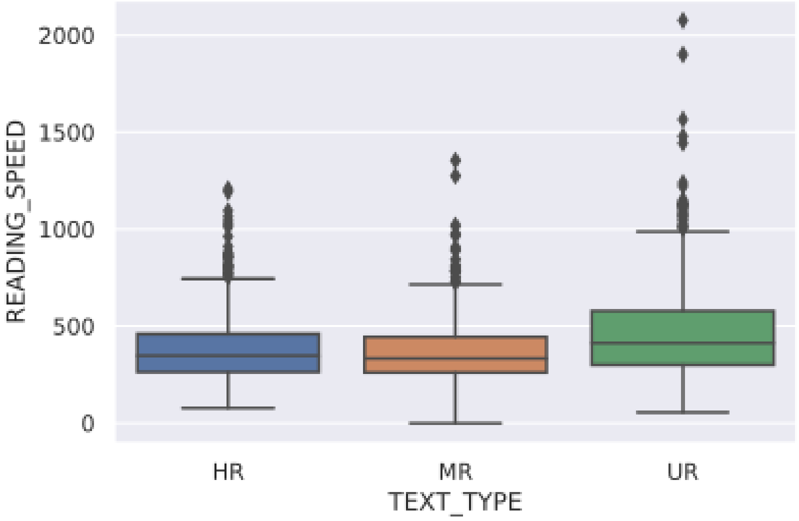

Figure S6. Boxplot of reading speed in wpm for each text type.

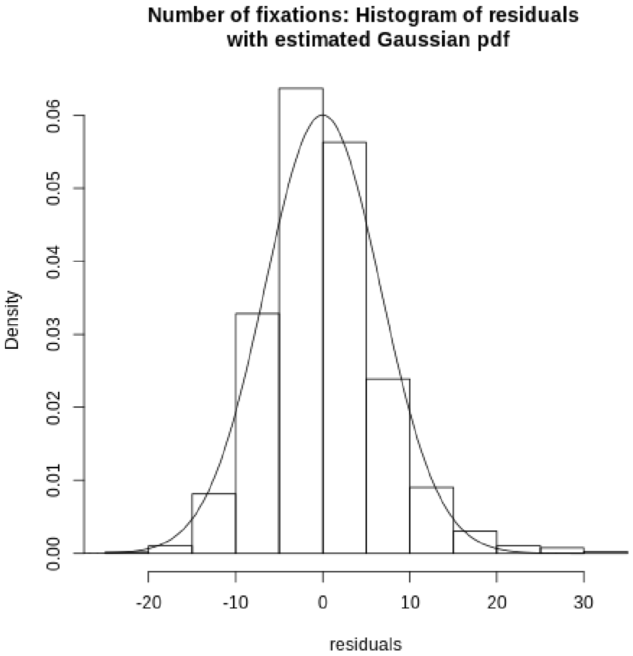

Figure S7. Histogram and estimated normal pdf for the LMMs residuals in modelling the effect of text type on the number of fixations per scanpath.

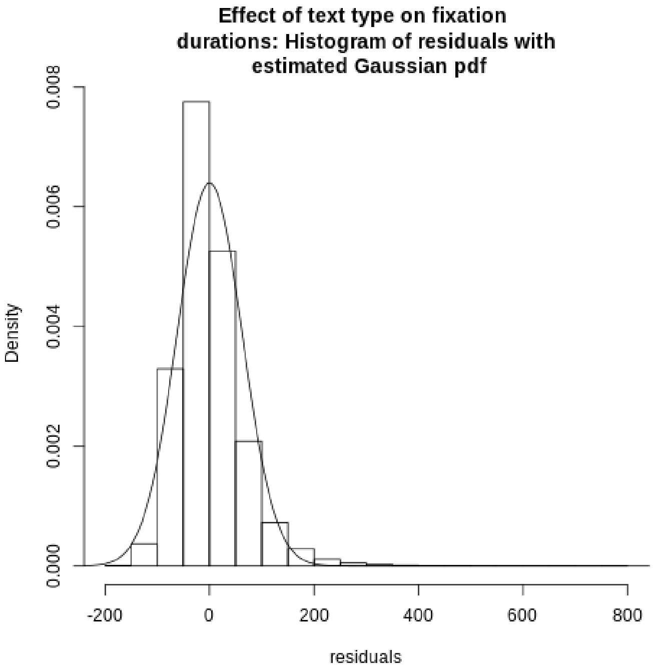

Figure S8. Histogram and estimated normal pdf for the LMMs residuals in modelling the effect of text type on fixation duration in msec.

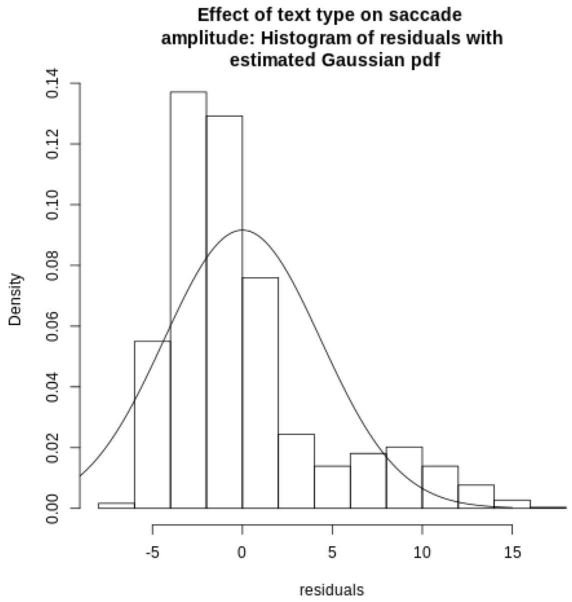

Figure S9. Histogram and estimated normal pdf for the LMMs residuals in modelling the effect of text type on saccade amplitude in degrees.

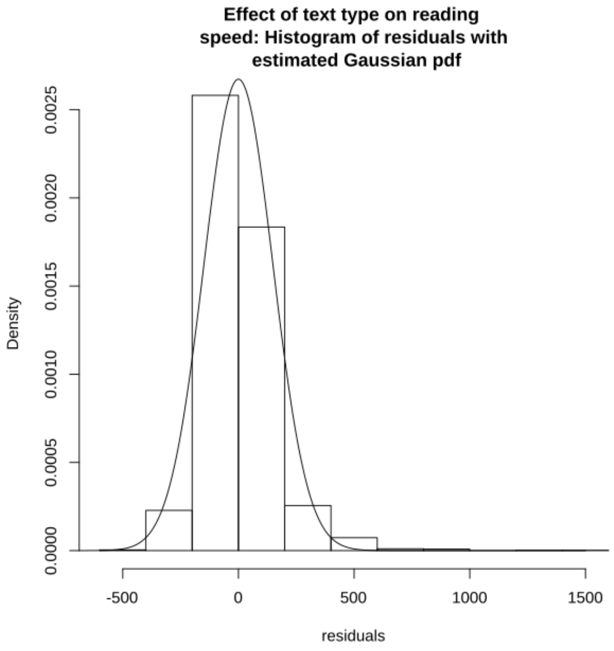

Figure S10. Histogram and estimated normal pdf for the LMMs residuals in modelling the effect of text type on reading speed in wpm.

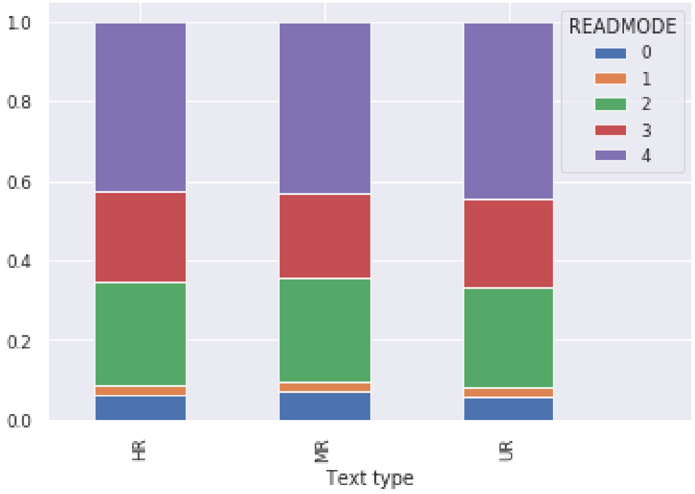

Figure S11. Sample distribution of *Read mode* for each text type. *Read mode* 0 corresponds to Bwd-, 1 to Bwd, 2 to Rfx, 3 to Fwd and 4 to Fwd+.

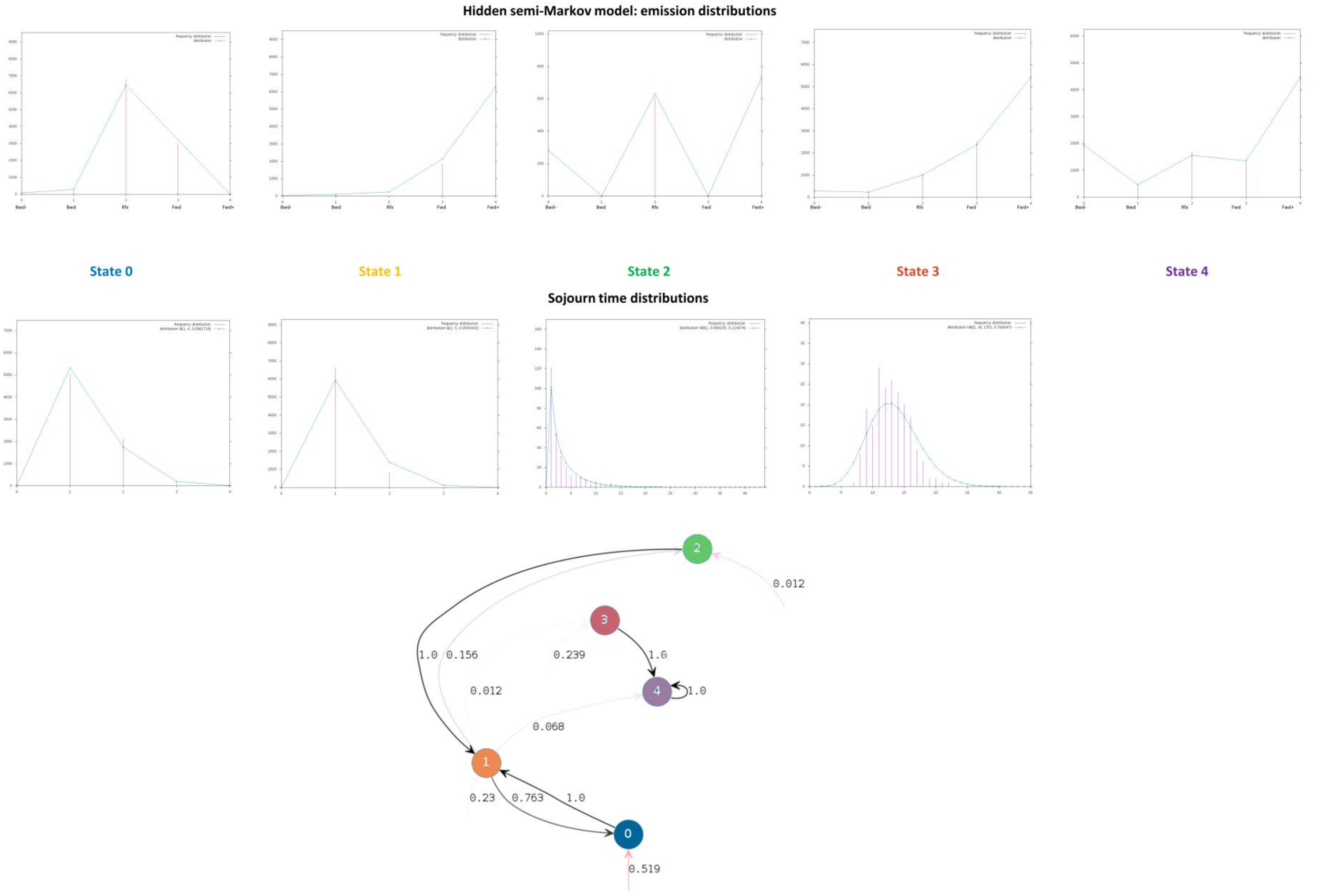
Hidden semi-Markov model: emission distributions
Bwd-
Bwd
Rfx
Fwd
Fwd+
State 0
State 1
State 2
State 3
State 4
Sojourn time distributions
0
1
2
3
4
1.0
0.156
0.239
0.012
0.068
0.23
0.763
0.519

Figure S12. Estimated HMSC parameters. Lines 1 and 2: estimated emission and sojourn state distributions for each state. States are in columns. Line 1: emissions distributions are defined as P(X_t=x | S_t=k) for the states k and Read mode values x. The latter are coded as follows: Bwd- / 0 / W_t < -1; Bwd / 1 / W_t = -1; Rfx / 2 / W_t = 0; Fwd / 3 / W_t = 1; Fwd+ / 4 / W_t > 1. State 4 is absorbing and has not estimated sojourn time distribution. Red vertical bars correspond to the number of observations (Line 1: Read mode, Line 2: sojourn durations) for the different states, which are restored by the Maximum A Posteriori principle. Green curves correspond to the distributions estimated by the EM algorithm, with their areas under curve rescaled by the number of observations. Line 3: estimated transition diagram between states. Vertices correspond to states and, arcs to transition probabilities above 0.01. The initial state distribution is represented by pink arrows pointing to possible initial states but issued from no other state.

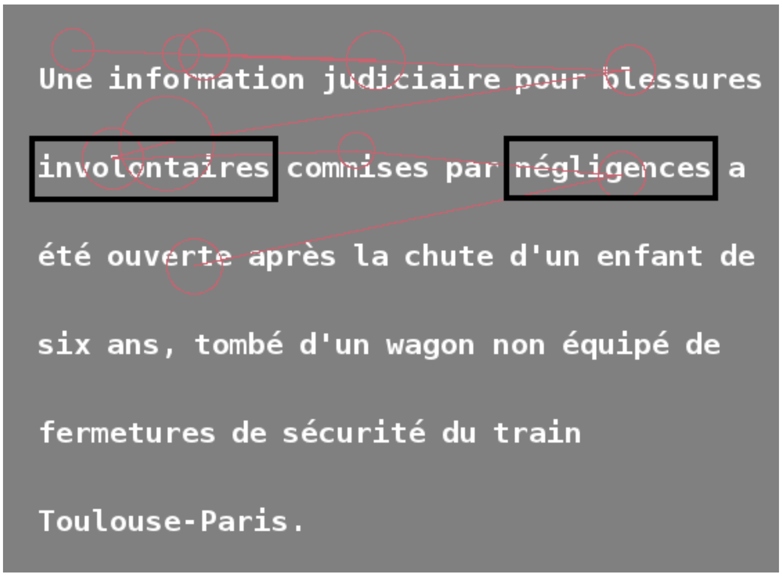

Figure S13. Scanpath of some UR text with phase restoration. Target topic is “Contemporary art”. Phase 3 (fast reading) is in red. Translation: “Judicial investigation for accidental injury due to negligence was opened after a six-year-old boy fell from a train joining Paris from Toulouse and lacking of secure locking mechanism”. The words framed in black (“accidental” and “negligence”) are the farthermost to target topic.

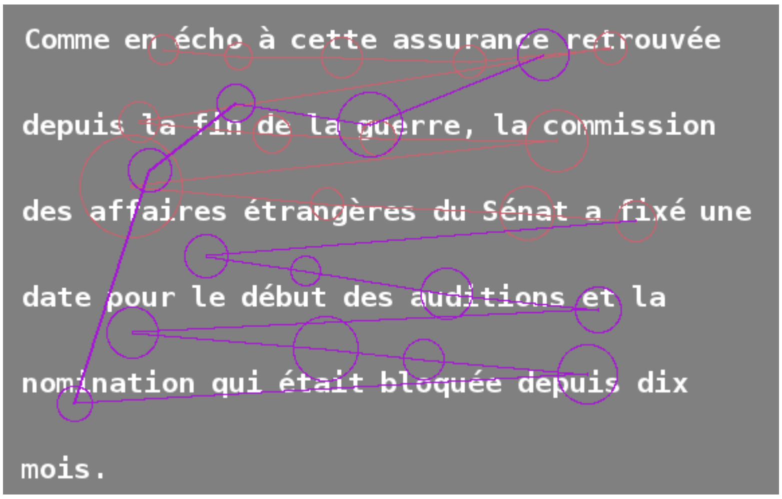

Figure S14. Scanpath of some MR text with phase restoration. Target topic is “Conflict in Irak”. Phase 3 (fast reading) is in red and Phase 4 (slow confirmation) in purple. Translation: “As if echoing the self-confidence regained since the end of the war, the Foreign Affairs committee of Senate set up a date to begin auditions and to proceed to nomination, which had been blocked for ten months”.

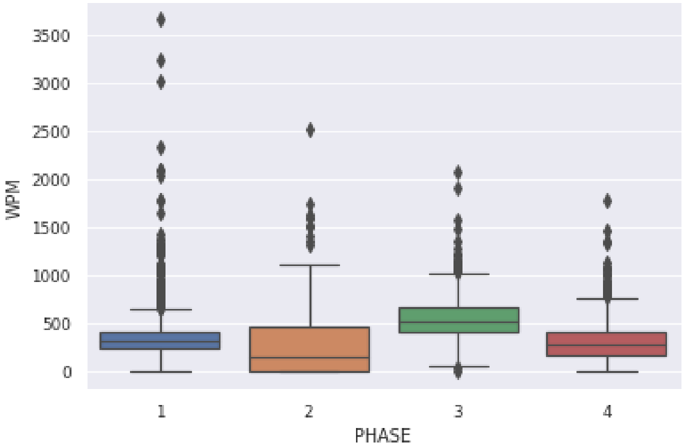

Figure S15. Boxplot of reading speed in wpm for each phase

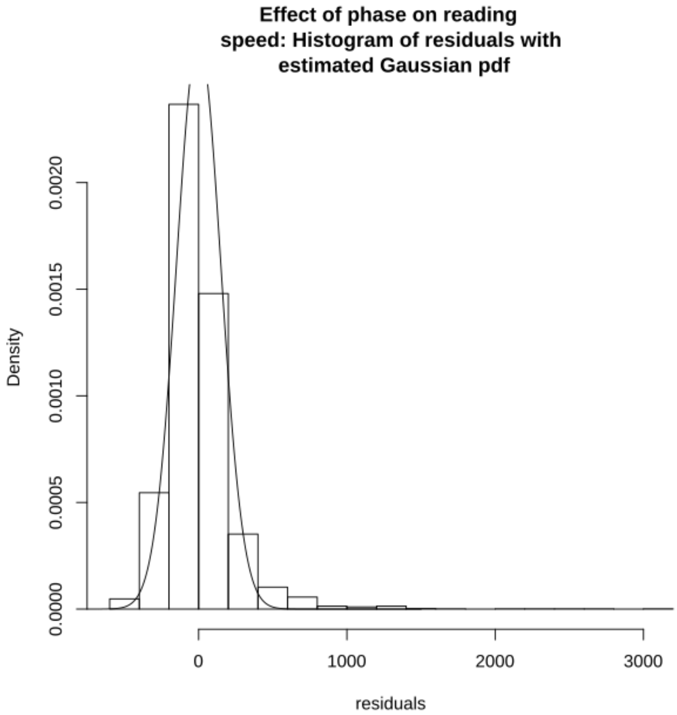

Figure S16. Histogram and estimated normal pdf for the LMMs residuals in modelling the effect of phase and text type on reading speed per scanpath.

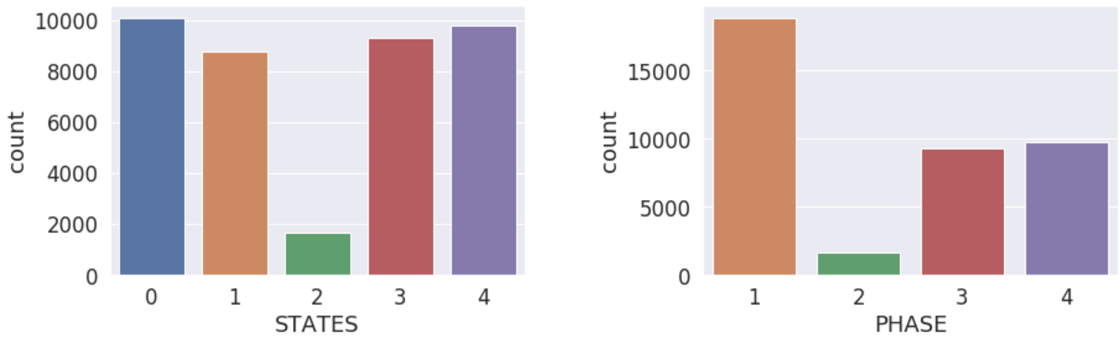

(a) (b)

Figure S17. (a) State sample distribution; (b) Phase sample distribution. Phase 0 (normal reading) is in orange, Phase 1 (information search) in green, Phase 2 (fast reading) in red and Phase 3 (slow confirmation) in purple.

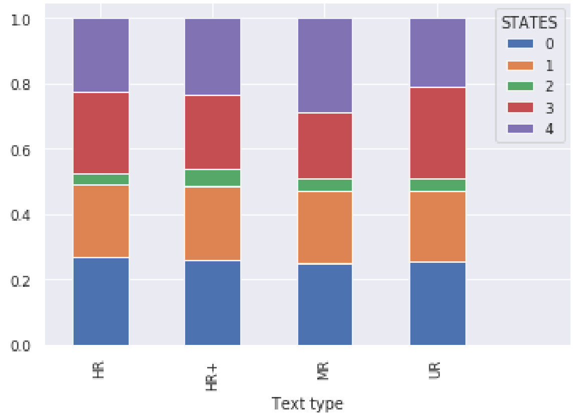

Figure S18. State sample distribution per extended text type.

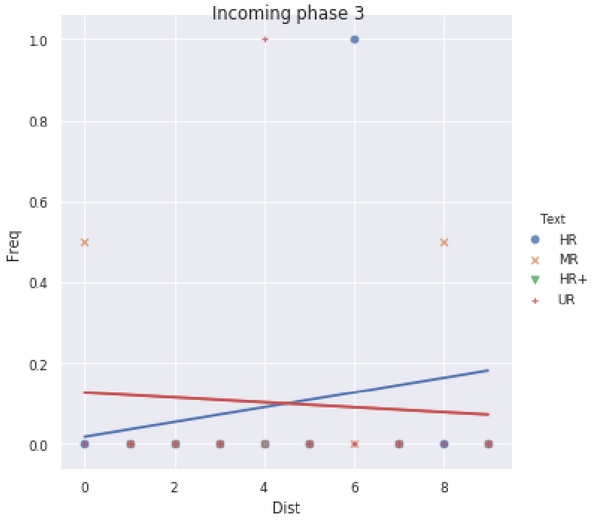

Figure S19. Relationship between distance to trigger words and frequencies of transitions arriving into phase 3 (fast reading).

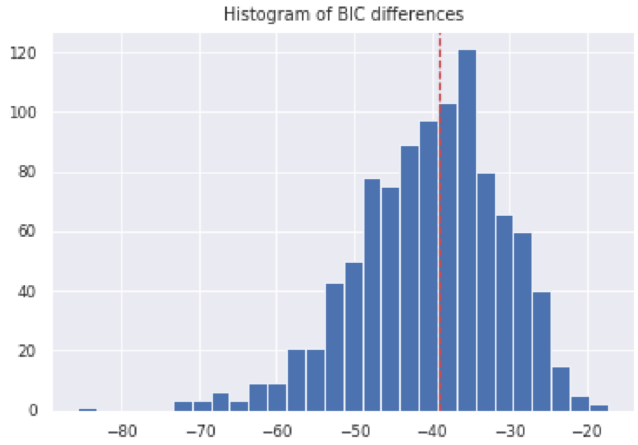

Figure S20. Histogram of BIC differences of alternative model M1 with null model M0 obtained by **constrained** permutations of phases under **linear** mixed models. The BIC difference corresponding to true data is represented by a dotted vertical line.

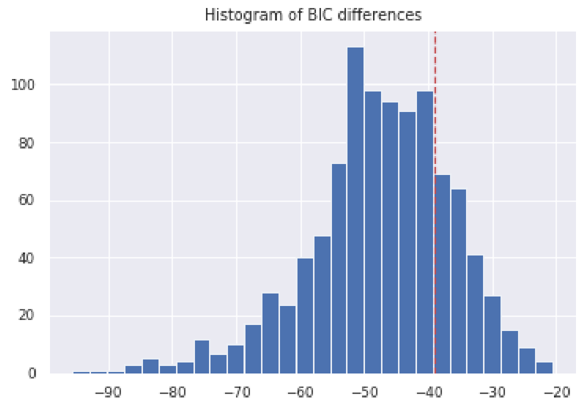

Figure S21. Histogram of BIC differences of alternative model M1 with null model M0 obtained by **free** permutations of phases under **linear** mixed models. The BIC difference corresponding to true data is represented by a dotted vertical line.

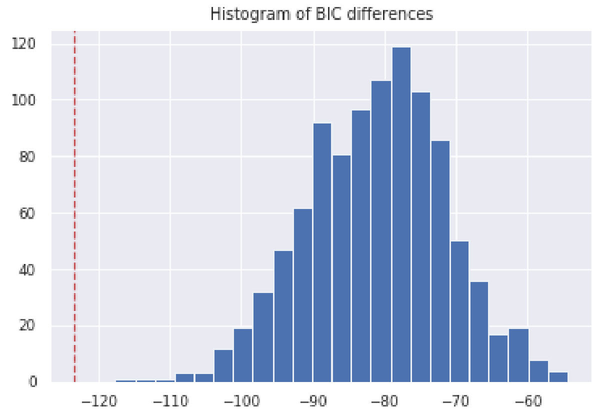

Figure S22. Histogram of BIC differences of alternative model M1 with null model M0 obtained by **constrained** permutations of phases under **generalized linear** mixed models. The BIC difference corresponding to true data is represented by a dotted vertical line.

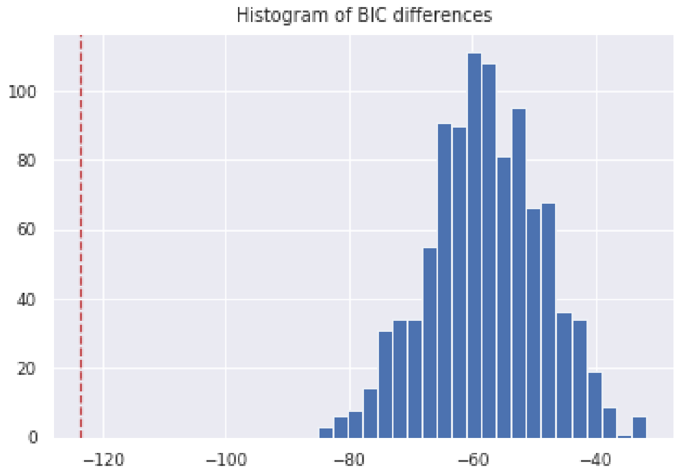

Figure S23. Histogram of BIC differences of alternative model M1 with null model M0 obtained by **free** permutations of phases under **generalized linear** mixed models. The BIC difference corresponding to true data is represented by a dotted vertical line.

Additional references used in this document

Yu, S. Z. (2010). Hidden semi-Markov models. *Artificial Intelligence*, 174(2), 215–243. doi: 10.1016/j.artint.2009.11.011
